# Archaeal and bacterial diversity in an arsenic-rich shallow-sea hydrothermal system undergoing phase separation

**DOI:** 10.3389/fmicb.2013.00158

**Published:** 2013-07-09

**Authors:** Roy E. Price, Ryan Lesniewski, Katja S. Nitzsche, Anke Meyerdierks, Chad Saltikov, Thomas Pichler, Jan P. Amend

**Affiliations:** ^1^Department of Earth Sciences, University of Southern CaliforniaLos Angeles, CA, USA; ^2^Department of Biological Sciences, University of Southern CaliforniaLos Angeles, CA, USA; ^3^Department of Molecular Ecology, Max Planck Institute for Marine MicrobiologyBremen, Germany; ^4^Geomicrobiology, Center for Applied Geosciences, University of TübingenTübingen, Germany; ^5^Department of Microbiology and Environmental Toxicology, University of California, Santa CruzSanta Cruz, CA, USA; ^6^Department of Geochemistry and Hydrogeology, University of BremenBremen, Germany

**Keywords:** microbial diversity, arsenic, hydrothermal, gradients, milos, phase separation

## Abstract

Phase separation is a ubiquitous process in seafloor hydrothermal vents, creating a large range of salinities. Toxic elements (e.g., arsenic) partition into the vapor phase, and thus can be enriched in both high and low salinity fluids. However, investigations of microbial diversity at sites associated with phase separation are rare. We evaluated prokaryotic diversity in arsenic-rich shallow-sea vents off Milos Island (Greece) by comparative analysis of 16S rRNA clone sequences from two vent sites with similar pH and temperature but marked differences in salinity. Clone sequences were also obtained for *aioA*-like functional genes (AFGs). *Bacteria* in the surface sediments (0–1.5 cm) at the high salinity site consisted of mainly *Epsilonproteobacteria* (*Arcobacter sp*.), which transitioned to almost exclusively *Firmicutes* (*Bacillus sp*.) at ~10 cm depth. However, the low salinity site consisted of *Bacteroidetes* (*Flavobacteria*) in the surface and *Epsilonproteobacteria* (*Arcobacter sp*.) at ~10 cm depth. *Archaea* in the high salinity surface sediments were dominated by the orders *Archaeoglobales* and *Thermococcales*, transitioning to *Thermoproteales* and *Desulfurococcales* (*Staphylothermus sp*.) in the deeper sediments. In contrast, the low salinity site was dominated by *Thermoplasmatales* in the surface and *Thermoproteales* at depth. Similarities in gas and redox chemistry suggest that salinity and/or arsenic concentrations may select for microbial communities that can tolerate these parameters. Many of the archaeal 16S rRNA sequences contained inserts, possibly introns, including members of the *Euryarchaeota*. Clones containing AFGs affiliated with either *Alpha-* or *Betaproteobacteria*, although most were only distantly related to published representatives. Most clones (89%) originated from the deeper layer of the low salinity, highest arsenic site. This is the only sample with overlap in 16S rRNA data, suggesting arsenotrophy as an important metabolism in similar environments.

## Introduction

Geochemical gradients often dictate microbial community structures, and metabolic processes directly influence these gradients. In hydrothermal systems, steep gradients generate redox disequilibria that can provide the necessary energy for a wide diversity of *Archaea* and *Bacteria*. Since their discovery in the 1970s, deep-sea hydrothermal environments have garnered much attention in this regard, with a number of studies linking geochemical composition, reaction energetics, and metabolic diversity (e.g., Flores et al., 2011; Meyer-Dombard et al., 2011). Their shallow-sea counterparts provide highly complementary research opportunities that are often overlooked. These systems are ubiquitous, readily accessible, and geochemically diverse; many exhibit geological, chemical, and biological characteristics similar to those found at deep-sea vents.

Shallow-sea and deep-sea hydrothermal systems also differ in several key ways (Dando et al., 1999; Pichler, 2005; Tarasov et al., 2005; Price et al., 2012). For example, the evolved discharging hydrothermal fluids in shallow-sea systems may have originally been seawater, meteoric water, or a mixture of the two. They often occur within the photic zone and thus provide the opportunity for both chemosynthetic *and* photosynthetic microbial metabolisms. They are most often associated with arc volcanism, which provides the heat source that drives hydrothermal circulation. Well known examples of shallow-sea hydrothermal systems are found near the Tabar-Feni (Pichler et al., 1999, 2006), the Aeolian (Italiano and Nuccio, 1991; Amend et al., 2003), the South Aegean (or Hellenic; Varnavas and Cronan, 1988; Dando et al., 1995), the Caribbean (McCarthy et al., 2005), and the Kurile-Kamchatka island arcs (Tarasov et al., 1990). In addition, some shallow-sea vent systems also occur in other areas related to faulting (e.g., Bahía Concepción; Forrest et al., 2005) or serpentinization reactions (e.g., New Caledonia; Cox et al., 1982). Many of these shallow-sea systems are sediment covered, allowing steep vertical and horizontal geochemical gradients to evolve as reduced, low pH, high temperature fluids mix with overlying seawater (e.g., Price et al., 2007). Across the geochemical gradients, “niches” of potential energy develop that constrain the resident microbial communities. While most shallow-sea hydrothermal fluids seem to be sulfur- and/or iron-rich, they can also be elevated in potentially toxic elements such as As, Sb, Cr, Pb, Cd, Cu, Zn, and Hg (Varnavas and Cronan, 1988; Koski et al., 2001; Pichler et al., 2006; Price et al., 2012).

Many seafloor hydrothermal systems are modified by phase separation (German and Von Damm, 2003; Von Damm et al., 2003), resulting in vent fluids that can vary drastically in salinity, from <6 to ~200% of seawater values (German and Von Damm, 2003). The resultant high and low salinity fluids differ markedly in the concentrations of many solutes, but the relation to microbial community structures has not been adequately considered. Previous investigations indicate that the vapor phase fluids may be enriched in important electron donors, such as H_2_, relative to the high salinity brine phase, and may contribute to stratification of microbial communities (Nakagawa et al., 2005; Nunoura and Takai, 2009). Arsenic is known to partition into the vapor phase (Pokrovski et al., 2002; Price et al., 2012). If the hydrothermal reservoir is enriched in arsenic and undergoing phase separation, this element can therefore be elevated in both high and low salinity discharging hydrothermal fluids. The highest As levels in a marine hydrothermal fluid were reported for the shallow-sea hydrothermal vent system in Palaeochori Bay, Milos Island, Greece (Price et al., 2012). There, high arsenic concentrations were reported for high and low salinity fluids; typically in the range of 30 μ M, but as high as 78 μ M in the low salinity fluids, suggesting As partitions into the vapor phase in this system.

The purpose of this investigation was to identify the dominant bacterial and archaeal lineages in Palaeochori Bay in an attempt to understand the link between geochemical gradients and microbial groups, including arsenic oxidizers. These relationships were investigated at two sites with similar pH and temperature, but different salinities.

## Site characteristics

**Figure 1:**
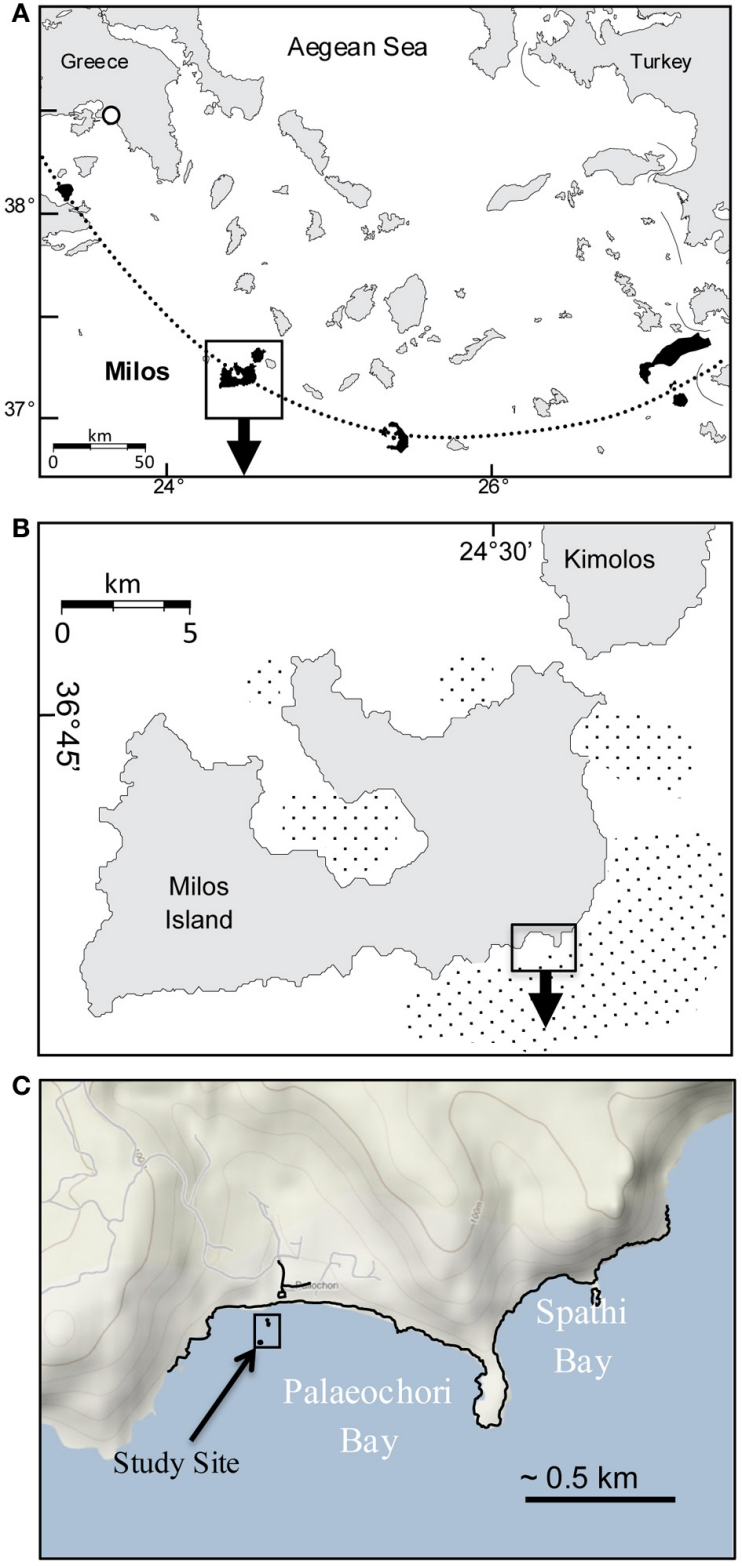
**(A)** Location of Milos Island (box) and other calc-alkaline volcanoes (shaded) along the Aegean island arc (dotted line). **(B)** Milos Island and the location of Palaeochori and Spathi Bays (box). Stippled offshore areas around the island are mapped gas emissions by echo sounding (Dando et al., 1995). **(C)** Location of our study site (box) in Palaeochori Bay.

**Figure 2:**
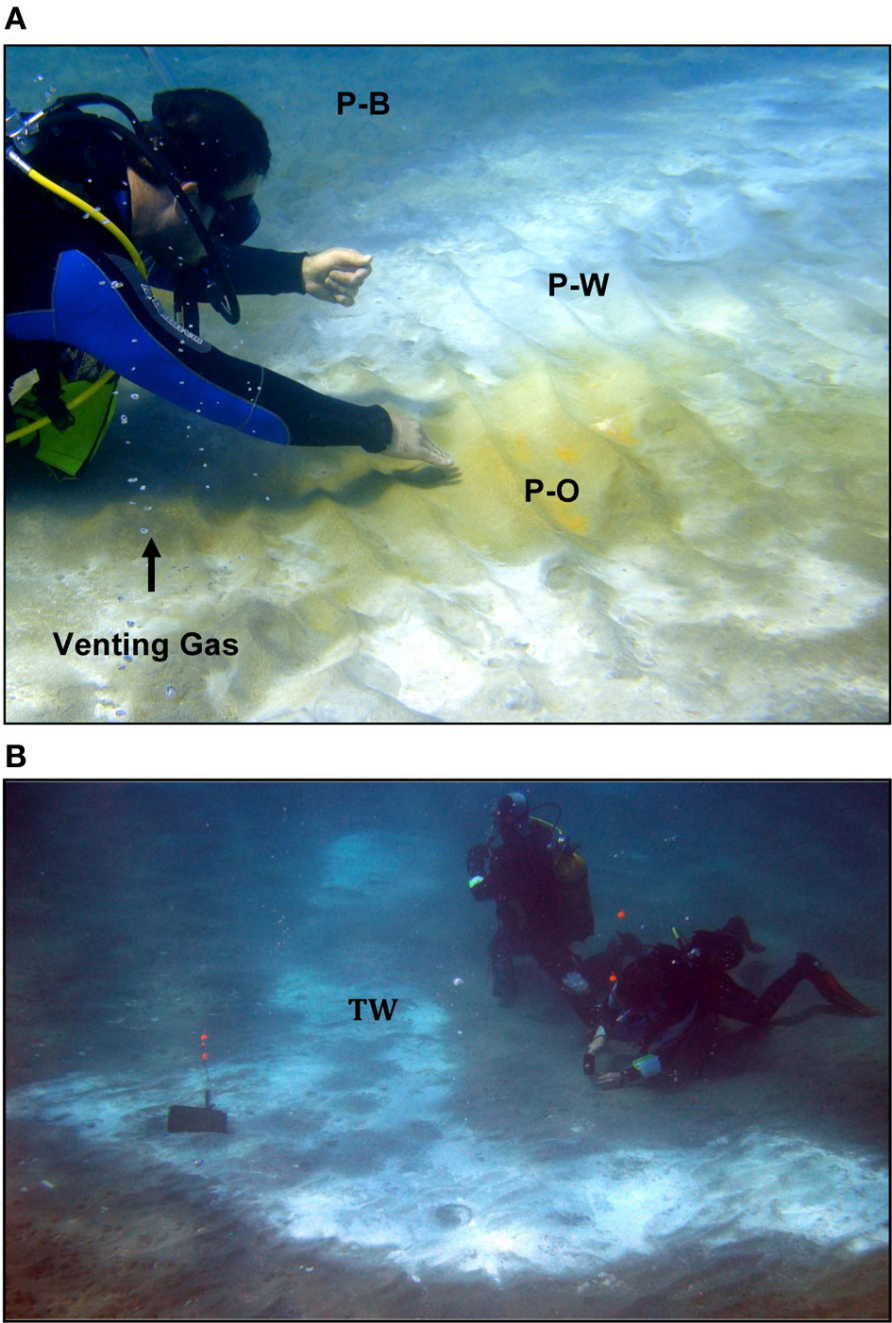
**(A)** Photograph of the RP site investigated in this study. P-O, Palaeochori orange; P-W, Palaeochori white; and P-B, Palaeochori background. Geochemical profiles and sediment cores were sampled at ~P-W. **(B)** Photograph of the TW site. Geochemical and microbiological samples for this study are indicated at the location of TW.

Gas discharge defines an ~35 km^2^ area of hydrothermal venting around Milos, making it one of the largest shallow-sea hydrothermal systems described to date (Figures 1A,B; Dando et al., 1995). The most intense venting occurs in Palaeochori Bay, where CO_2_-rich gases and hydrothermal fluids discharge through sand (Figures 1A–C, 2). Nearby—but little explored—Spathi Bay, to the east, also features abundant hydrothermal venting, although free gas emissions are less abundant. The high salinity hydrothermal fluids in Palaeochori Bay were slightly acidic (pH ~5), sometimes highly sulfidic (up to 3 mM), and warm (ambient to ~110°C; Stüben and Glasby, 1999; Valsami-Jones et al., 2005; Price et al., 2012).

The occurrence of orange, white, or brown microbial mats and hydrothermal precipitates on the seafloor sediments are a major feature of the hydrothermal system. The different colors correlate to different sediment temperatures, where the hottest areas (>90°C) are typically covered by bright yellow native sulfur or orange-colored arsenic sulfide deposits (Figure 2A). White mats make up the largest areas of hydrothermal venting throughout the bay, and display mid-range temperatures (e.g., ~45–85°C). In the lower temperature areas (~30–35°C), brown colored manganese and/or iron oxide deposits are present (Figure 2A).

Several microbiological investigations were carried out for the high salinity sites in Palaeochori Bay, although none to date targeted low salinity environments. They included culture-dependent and molecular-based microbiological studies, as well as the isolation of several new bacterial and archaeal species (Jochimsen et al., 1997; Dando et al., 1998; Brinkhoff et al., 1999; Sievert et al., 1999, 2000a,b; Arab et al., 2000; Sievert and Kuever, 2000; Schlesner et al., 2001; Bayraktarov et al., 2013). One study investigated the microbial ecology and arsenic functional genes in lower temperature 0–6 cm pooled sediments at the same high salinity site investigated here (Nitzsche, 2010). Results indicated a high diversity in the bacterial community sequences of *Gamma*-, *Delta*-, and *Epsilonproteobacteria*, *Bacteroidetes*, and *Cyanobacteria*. Closest cultivated relatives were associated with photo- and chemotrophic lifestyle and seemed mostly to be involved in sulfur cycling. Archaeal sequences predominantly affiliated with *Thermoplasmatales* or *Thermococcales*. Screening for arsenic functional genes indicated affiliation with either *Alpha-* or *Betaproteobacteria*, although affiliation with *Gammaproteobacteria* was noted. These studies indicated that metabolisms based on sulfur, either oxidation of H_2_S or reduction of SO^2−^_4_, may be dominant at the high salinity sites, although dissimilatory iron reducing (DIR) *Bacteria* are abundant in some FeIII-rich sediments. They also suggest that both heterotrophic and chemotrophic metabolisms are possible, with a range from mesophilic to hyperthermophilic functioning, and that arsenite oxidase genes are present at the high-salinity, lower temperature sites. This study expands on these investigations by studying the microbial ecology at greater sediment depth than the previous investigations and for the first time compares community structure between a high salinity and low salinity site.

## Materials and methods

### Site description

Two sites in Palaeochori Bay [Rocky Point (RP) and Twinkie (TW)] were investigated in this study; both are ~100 m offshore in ~4.5 m deep water (Figures 1C, 2A,B). Preliminary data indicate nearly identical pH and temperature profiles, although the RP site was demonstrably more saline than the TW site. The RP site features concentric rings of colored sediments, with orange in the center, transitioning to white and then brown (Figure 2A). The TW site is located ~8 m due east of the RP site and consisted primarily of white mats with some gas bubbles at the north side of the patch. Samples for this study were collected from the white areas from both sites (“P-W” in Figure 2A and “TW” in Figure 2B).

#### Field sampling protocols

Pore fluids and sediments for geochemical and microbiological analyses were collected by SCUBA. Sediments were cored with polycarbonate tubes (Arthur Krüger GmbH) and capped underwater with rubber end caps. Pore fluids were either collected *in situ* into a BD™ (Becton, Dickinson and Company) 60 mL syringe from a tube inserted into the sediments, or extracted through holes in the core tube once onshore using 10 mL syringes attached to rhizons (long filters which can be inserted into the core; Rhizosphere) following Seeberg-Elverfeldt et al. (2005). Sediment cores were sliced for subsequent microbiological analyses. Each core slice was aseptically placed into sterile bags and frozen on dry ice. Samples were then shipped on dry ice back to the laboratory and kept frozen at −80°C.

Samples for free gas geochemistry were obtained by inverting a serum bottle under water and holding it over the streaming gas bubbles. Once the gas had completely filled the bottle, it was capped and crimped underwater with little to no seawater inside the bottle. Pore fluids for dissolved gases were sampled at 10 cm depth in the sediments by inserting a tube into the sediments, and filling a syringe. Subsequently, these pore fluids were put into N_2_-filled 60 mL serum bottles until a reverse meniscus formed, then capped and crimped.

#### Geochemistry

Temperatures were measured *in situ* using a temperature probe in a custom-built underwater housing (constructed at the Max Planck Institute for Marine Microbiology, Bremen, Germany). The pH was measured on shore using a WTW pH meter 3210 with Mic-D electrode with temperature compensation. Analytical uncertainties were approximately ±0.1°C for temperature and ±0.1 for pH. Pore fluid concentrations of Fe^2+^, NO^−^_3_, NO^−^_2_, and NH_3_ were analyzed in the field using a HACH spectrophotometer. Samples for free (CO_2_, O_2_, N_2_, He, H_2_, CO, and CH_4_) and dissolved (He, H_2_, O_2_, N_2_, CO, CH_4_, and CO_2_) gases were shipped on ice to the Istituto Nazionale di Geofisica e Vulcanologia, Palermo (Italy), for analysis. Pore fluid samples for anion analysis (Br, Cl, and SO^2−^_4_) were filtered in the field (0.2 μm), placed on dry ice, and kept frozen until measurement in the laboratory. Samples for analysis of major seawater cations (Na, Ca, K, B, Sr), minor elements (Si, Ba, Mn, Fe), and trace elements (Li, Rb, Cs) were preserved in the field by filtering (0.2 μm) and acidification with 0.1% ultrapure HNO_3_.

### Laboratory protocols

#### Geochemistry

Major anions were analyzed using a Dionex ion chromatography system, whereas major cations were measured by inductively coupled plasma-optical emission spectrometry (ICP-OES; Perkin-Elmer Optima 3300). Lithium, rubidium, and cesium were measured by inductively coupled plasma-mass spectrometry (ICP-MS; High-resolution double-focusing ICP-MS Thermo Finnigan Element 2). Free (CO_2_, O_2_, H_2_, CO, and CH_4_) and dissolved (H_2_, O_2_, CO, CH_4_, and CO_2_) gases were measured on a Perkin Elmer 8500 gas chromatograph equipped with a double-detector (TCD-FID) using argon as carrier gas (Italiano et al., 2009).

#### Microbiology

***DNA extraction, PCR, and Cloning***. Bulk environmental (genomic) DNA was extracted from ~0.5 g of sediment according to the protocol outlined in the MPBio FastDNA® spin kit, and stored at −20°C. Amplification of 16S rRNA genes for both *Archaea* and *Bacteria* was performed by polymerase chain reaction (PCR) using primer sets 27F/1492R and 21F/1391R, respectively (Lane, 1991), on a Hybaid PCR express thermocycler. A total reaction volume of 20 or 50 μ L was prepared, containing 1× PCR buffer, 0.25 mM each dNTPs, 0.5 μ M each forward and reverse primer, 0.5–2 μ L DNA template depending on sample concentration, and 5 U Taq DNA polymerase (5 PRIME). DMSO was added to some samples to increase reaction yield. The final volume was adjusted with autoclaved milli-Q water. The amplification was initiated with denaturation at 95°C for 5 min, followed by denaturation (at 95°C for 1 min), annealing (at 55°C for *Archaea*, 52°C for *Bacteria*, for 1 min), and elongation (at 72°C for 1 min) for 30–35 cycles. A final extension step was carried out at 72°C for 10 min. Agarose gel electrophoresis was performed to verify amplification.

Degenerate primer sets used for arsenite oxidase functional gene (*aioA*-like) amplification were AOX-F-A2 (5′-TGC ATCGTCGGCT GYGGNTAY-3′) and AOX-R-E2 (5′-TTCGGAGTTATAG GCCGGNCKRTTRTG-3′) (Zargar et al., 2012) on a Hybaid PCR express thermocycler. These primers target the *aoxB* gene sequence of the arsenite oxidase operon and typically result in ~670 bp product. A total reaction volume of 50 μL was prepared, containing 1× PCR buffer, 0.25 mM each dNTPs, 0.5 μ M each forward and reverse primer, 0.5–2 μ L DNA template depending on sample concentration, and 5 U Taq DNA polymerase (5 PRIME). DMSO was added for some samples to increase reaction yield, and the final volume was adjusted with autoclaved milli-Q water. The amplification was initiated with denaturation at 95°C for 1 min, followed by denaturation (at 95°C for 0.5 min), annealing (at 57°C for 0.5 min), and elongation (at 72°C for 1 min) for 35 cycles. A final extension step was carried out at 72°C for 5 min. Agarose gel electrophoresis was performed to verify amplification.

PCR products were cloned separately using the pCR4-TOPO® Plasmid Vector Kit with One Shot TOP10 chemically competent *Escherichia coli* cells. Volumes of 25, 50, and 100 μ L of each transformation reaction were spread on selective LB agar plates containing ampicillin and incubated overnight at 37°C. White colonies were transferred with sterile toothpicks into 2.5 mL, 96 well microtiter plates containing a final volume of 200 μ L of LB medium. After an overnight incubation at 37°C, 100 μ L aliquots of liquid cultures were pipetted into a 96 well standard microplate with 20 % glycerol and shipped frozen to Beckman Coulter for sanger sequencing.

***16S rRNA gene clone library analysis***. Forward and reverse sequence fragments were trimmed and assembled using Geneious version 5.6.3, created by Biomatters (http://www.geneious.com/). Contigs were checked for chimeras using Mothur (Schloss et al., 2009) and Bellerophon (Huber et al., 2004). Sequences were classified using Mothur's Bayesian classifier and the RDP training set version 9 (Wang et al., 2007). Sequences were also aligned, compared, and clustered into operational taxonomic units (OTUs) using the default settings in Mothur. Representative OTUs at the 97 % sequence identity were selected and used to search for similar sequences in NCBI's nr/nt database using nucleotide BLAST (http://blast.ncbi.nlm.nih.gov/Blast.cgi). Organisms with complete genomes, closest cultured representatives, or sequences isolated from relevant hydrothermal or arsenic-rich environments were added as references to the 16S rRNA and *aioA*-like phylogenetic trees. The 97% OTU representatives and the database reference sequences where realigned in Mothur, and imported into Geneious. The PHYML plugin (Guindon and Gascuel, 2003) was used to construct a maximum likelihood tree for bacterial and archaeal sequences.

Several richness and evenness calculations were performed using their respective commands in Mothur. These include calculations for rarefaction curve data, Sobs (the observed richness reported, OTU), chao (the Chao1 estimator for richness), and the simpsoneven and shannoneven (both index-based measures of evenness), all at 97% cutoff.

For *aioA*-like gene sequences, assembly and vector trimming was also done in Geneious. Trimmed sequences were searched against Ref_Seq using BLASTx. Sequences that hit arsenite oxidase subjects with an *e*-value lower than e-40 were used to build the tree. Selected bacterial *aioA*-like genes where taken from Genbank and also added to the tree for reference. Sequences were aligned and compared in Geneious using both nucleotide and amino acid translation. The *aioA* tree was built using PHYML.

### Submission of nucleotide sequence and accession numbers

Sequence data for 16S rRNA bacterial and archaeal clone libraries were submitted to GenBank under accession numbers KF278514-KF278557 and KF278485-KF278513, respectively. Partial nucleotide sequences for the arsenite oxidase genes were submitted under accession numbers KF303544-KF303564.

## Results

### Geochemistry

**Figure 3:**
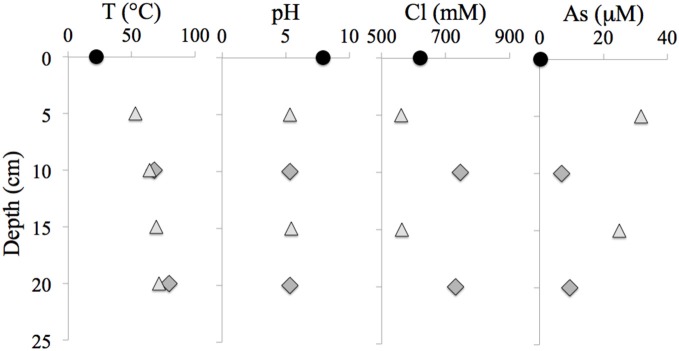
**Depth profiles of temperature, pH, chloride and arsenic concentrations at the RP and TW sites**. Note (1) similarities between the RP and TW site with respect to T and pH, and (2) differences between these sites with respect to Cl and As concentration. Seawater values are indicated with a filled circle, RP data are indicated with a dark gray filled diamond, and TW data are indicated with a light gray filled triangle.

Table 1 presents the geochemical data, including temperature, pH, Fe^2+^, NO^−^_3_, NO^−^_2_, NH_3_, and major and minor elements, collected from RP and TW sites. For comparison, seawater values, as well as a depth profile from a control site, were included. Temperature and pH were very similar between sites, but Mg^2+^ and SO^2−^_4_ concentrations were depleted in both RP and TW samples. Furthermore, major cations, such as Na, Ca, K, B, and Sr, were enriched in the RP samples, but depleted in the TW samples, relative to seawater. Major anions, such as Cl and Br, are also enriched in RP samples but depleted in TW samples, relative to seawater. Most minor elements, including Si, Li, Rb, Fe, and Ba, were enriched in both fluids, although only slightly so in the TW fluids. Mn was only enriched in the RP samples. Arsenic concentrations followed a different pattern compared to all other elements. Both sites were highly enriched in this element, but in this case, the TW site was much more so, with a maximum concentration of 31.7 μ M compared to 9.4 μ M at RP. Depth profiles of temperature, pH, and concentrations of Cl and As are also shown in Figure 3. Note that the values of temperature and pH for RP and TW were similar with depth, but values for Cl and As differed markedly between sites (Figure 3). Table 2 presents the dissolved and free gas data collected for this study. Dissolved gases were sampled at 10 cm depths at equivalent temperature and pH. Both RP and TW free gas was mostly CO_2_, followed by N_2_, O_2_, H_2_, CH_4_, He, and CO, although H_2_S concentrations in the free gas phase were not analyzed. Dissolved gases followed a similar trend.

**Table 1:** **Major physicochemical parameters, major and minor elements in hydrothermal fluids collected from Paleochori Bay hydrothermal system**.

**Site name**	**Field data**								**Major elements**									**Minor elements**						
	**Depth (cm)**	**Temp. (°C)**	**pH**	**Fe^2+^ (mM)**	**NO^−^_3_ (mM)**	**NO^−^_2_**	**NH_3_ (mM)**	H_2_S^**^	**Mg (mM)**	**Na (mM)**	**Ca (mM)**	**K (mM)**	**B (mM)**	**Sr (mM)**	**Br (mM)**	**Cl (mM)**	**SO_4_ (mM)**	**Si (mM)**	**Li (mM)**	**Rb (uM)**	**Fe (uM)**	**Mn (uM)**	**As (uM)**	**Ba (uM)**
Seawater	n.a.^*^	22.1	7.9						69.8	590.5	7.9	23.0	0.4	0.1	0.9	620.3	34.3	0.0	0.2	31.3	b.d.	0.0	0.4	b.d.
Control pore fluids	5	22.1	7.4					6	66.8	581.4	7.1	21.7	0.4	0.1	0.9	638.0	32.8	0.0	0.1	30.7	0.5	b.d.	0.2	0.0
Control pore fluids	15	23	7.4	0.024	0.06	n.a.	n.a.	42	68.6	583.3	7.5	22.8	0.4	0.1	0.9	640.1	33.0	0.1	0.1	30.8	29.5	b.d.	0.3	0.1
Rocky Point	10	67.9	5.3	n.a.	0.28	0.7	0.4	257	55.3	703.7	16.6	60.7	1.7	0.1	0.9	744.9	20.8	1.8	3.2	231.8	11.0	27.2	6.9	1.4
Rocky Point	20	79.4	5.3	n.a.	0.30	0.7	0.5	246	56.0	696.3	16.3	59.5	1.7	0.1	0.9	731.2	20.8	1.8	3.1	227.3	8.2	25.9	9.4	1.3
Twinkie	5	52.8	5.3	0.014	0.40	1.4	0.2	253	61.2	507.0	6.5	21.8	0.5	0.1	0.8	560.5	27.5	1.1	0.3	39.7	15.8	b.d.	31.7	0.6
Twinkie	10	64.3																						
Twinkie	15	69.6	5.4	0.005	0.38	0.8	0.1	286	59.8	507.6	6.2	21.2	0.5	0.1	0.8	563.4	29.9	1.1	0.3	39.2	8.3	b.d.	25.0	0.6
Twinkie	20	71.5																						

* *n.a. or empty cell, not available; b.d., below detection*.

** *Note: H_2_S data are from Druschel et al., unpublished data*.

**Table 2:** Free and dissolved gas collected for this study^*^.

**FREE**	**CO_2_ (%)**	**O_2_ (%)**	**N_2_ (%)**	**He (ppm)**	**H_2_ (ppm)**	**CO (ppm)**	**CH_4_ (ppm)**
RP	92.5	0.13	0.67	7.0	11450	0.7	916
TW	93.5	0.39	1.2	11.7	14635	2.2	1890.0
**[mL(STP)/L^#^]**							
**Dissolved**	**CO**_2_	**O_2_**	**N_2_**	**He**	**H**_2_	**CO**	**CH**_4_
RP	50.9	0.0256	2.4	^**^bdl	0.0077	0.00029	0.0058
TW	245.9	0.0109	3.2	0.00027	0.0004	0.00027	0.0303

* *Note: TW free and RP and TW dissolved gases are averages (n = 3; free gas for RP was only collected once)*.

** *bdl, below detection limit*.

# *Dissolved gas data reported as mL of gas at standard temperature and pressure per liter of water*.

### Microbiology

#### Richness and evenness

**Figure A1:**
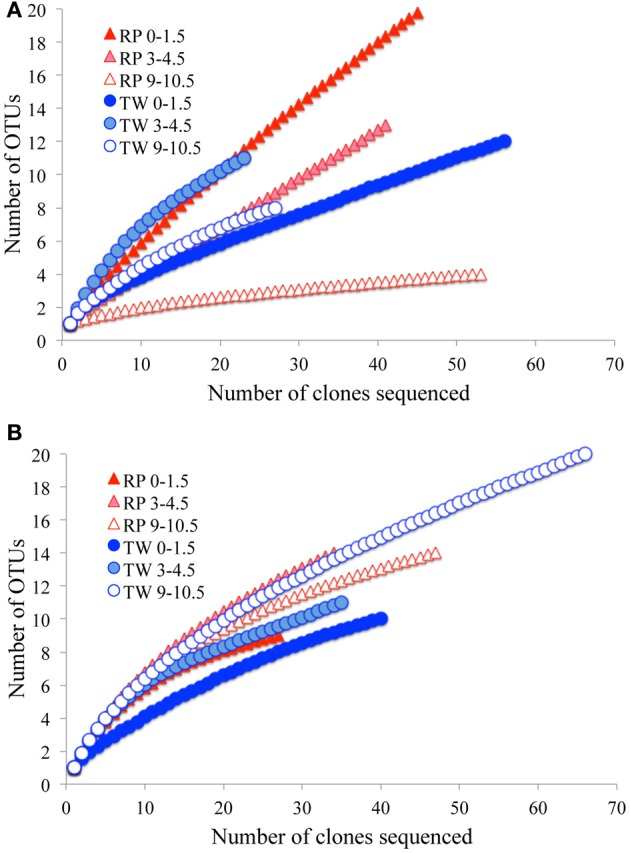
**Rarefaction curves showing 16S rRNA OTU richness for each of the sampling sites and depths for this study**. Curves were calculated using the furthest neighbor approach with a 97% sequence similarity cutoff. Data shown are for *Bacteria* **(A)** and *Archaea* **(B)**.

The total number of clones generated for *Bacteria* per site is as follows: RP 0–1.5 (51), RP 3–4.5 (41), RP 9–10.5 (53), TW 0–1.5 (56), TW 3–4.5 (23), and TW 9–10.5 (27). The total number of clones generated for *Archaea* per site was: RP 0–1.5 (27), RP 3–4.5 (34), RP 9–10.5 (47), TW 0–1.5 (40), TW 3–4.5 (35), and TW 9–10.5 (66). Rarefaction analyses of the 16S rRNA gene sequences for *Bacteria* (Figure A1A) and *Archaea* (Figure A1B) indicated that the RP 0–1.5 sample displayed the highest diversity, with 22 OTUs out of 51 sequences. The steep slope for the RP 0–1.5 sample indicates that a large fraction of the species diversity remains to be discovered. The same is true for the RP 3–4.5 layer, which had 13 OTUs out of 41 sequences. The RP 9–10.5 sample had the lowest bacterial diversity for all samples analyzed. However, the TW 0–1.5 sample had the lowest bacterial diversity of all TW samples, with 12 OTUs out of 56 sequences. The TW 9–10.5 layer diversity was similar to TW 0–1.5, only slightly more diverse, and a slight development of an asymptotic curve. The TW 3–4.5 layer had the highest bacterial diversity of all TW samples, with a slope similar to the RP 0–1.5 sample. The RP site showed a clear decrease in bacterial diversity vs. depth, although the TW site did not follow this pattern. Hydrothermal environments, compared with many other marine environments, are often dominated by relatively few taxa (Polz and Cavanaugh, 1995; Schrenk et al., 2004). Therefore, in theory, fewer clones may be necessary for adequate sampling and evaluation of diversity. However, it is important to note that these curves may be most useful for assessing relative differences in the diversity of major groups between sites and depths.

For the archaeal OTUs, both sites showed increasing diversity vs. depth (Figure A1B). Archaeal OTU diversity for the TW 0–1.5 layer was the lowest, sequences from TW 3–4.5 slightly more so, with the TW 9–10.5 layer having the highest diversity. The RP 0–1.5, RP 3–4.5, and RP 9–10.5 samples followed a similar trend, with increasing archaeal diversity vs. depth. Each of the archaeal rarefaction curves level off to the right of the plot, suggesting the archaeal diversity may have been sampled adequately.

Richness indices for *Bacteria* and *Archaea* for each site vs. depth are presented in Table 3. The Chao1 index indicated that generally the bacterial communities were more diverse in the surface layers of the RP site relative to any other sample, and both RP and TW bacterial richness decreased with depth. Both the Shannon index and Simpson index indicated that the most diverse samples were RP 0–1.5 and TW 3–4.5, and that the RP site bacterial richness decreases vs. depth, whereas the TW site has the highest bacterial richness in the 3–4.5 depth sample. Each of these three indices generally indicated that archaeal richness was highest in the deepest samples with overall richness increasing vs. depth.

**Table 3:**
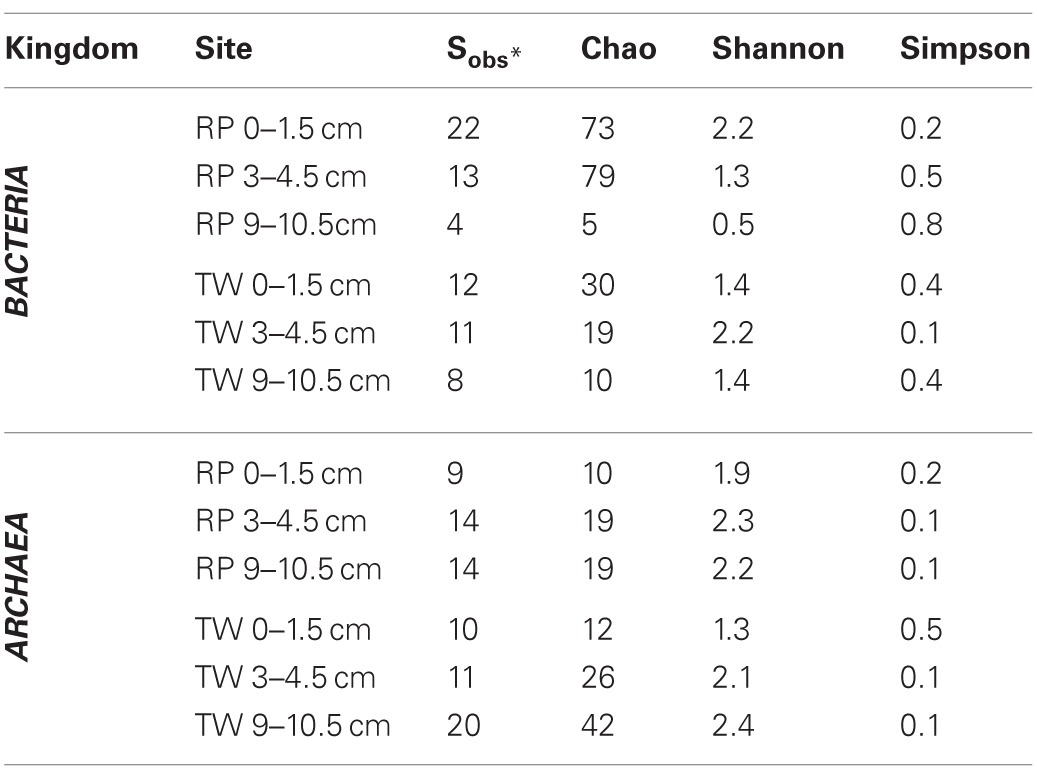
**Microbial diversity richness indices for bacterial and archaeal depth profiles from RP and TW sites in Palaeochori Bay**.

^*^Sobs, number of OTUs identified.

#### Bacterial affiliations

**Figure 4:**
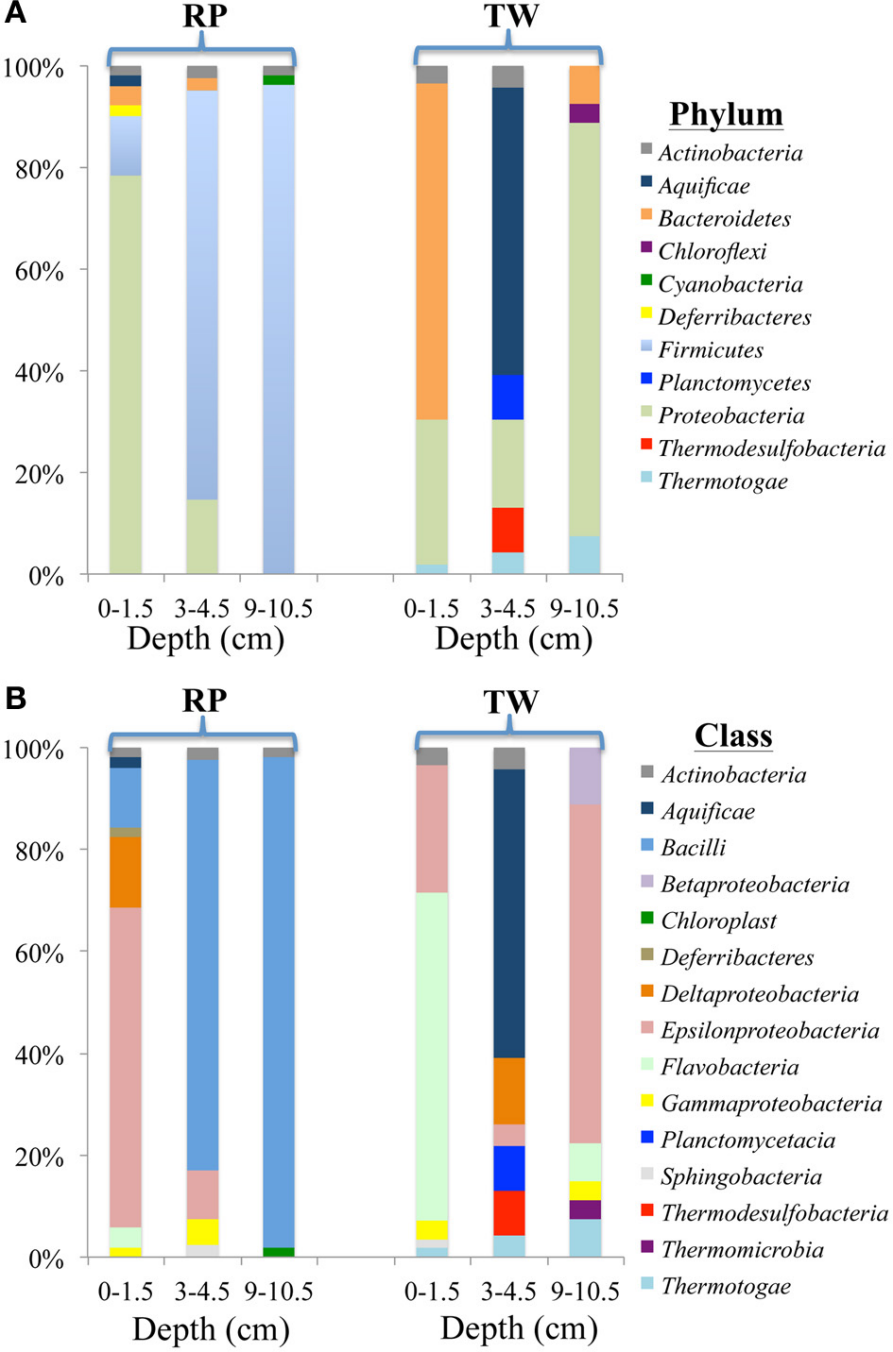
**Diversity and distribution of clones within bacterial phylum (A) and class (B) clone libraries for three depths of RP and TW sediment layers investigated in this study**. Each color represents the percentages of each taxon in the total respective clone library, as determined by screening of full length 16S rRNA gene sequences. Depths are 0–1.5, 3–4.5, and 9–10.5 cm, and indicated at the bottom of site designation.

The diversity and distribution of bacterial 16S rRNA clones at the phylum (Figure 4A) and class (Figure 4B) levels were investigated for three depths at RP and TW. The RP 0–1.5 cm sediments were dominated by the phylum *Proteobacteria* (40 out of 51 clones), followed by representatives from the *Firmicutes* (6), *Bacteroidetes* (2), *Actinobacteria* (1), *Aquificae* (1), and *Deferribacteres* (1) (Figure 4A). Within the *Proteobacteria*, the *Epsilon* class dominated (Figure 4B), with nearly all clones identified as *Arcobacter spp*. Minor classes include *Deltaproteobacteria* (7 clones), *Bacilli* (6), and 1–2 clones each affiliated with *Actinobacteria*, *Aquificae*, *Flavobacteria*, and *Deferribacteres* (Figure 4B). In the 3–4.5 cm layer, the *Firmicutes* phylum dominated (33/41), followed by *Proteobacteria* (6), and one clone each of *Bacteroidetes* and *Actinobacteria* (Figure 4A). The *Firmicutes* consisted almost entirely of *Bacillus spp*. (100% confidence); the *Proteobacteria* were composed of the *Epsilon* (4) and *Gamma* classes (2) (Figure 4B). The deepest layer investigated at RP (9–10.5 cm) was also dominated by the *Firmicutes* phylum (51/53; Figure 4A), with 47 clones identified as *Bacilli* (Figure 4B), predominantly within the genus *Bacillus*.

At TW in the 0–1.5 cm layer, *Bacteroidetes* dominated (37/56; Figure 4A), with 36 of those affiliating with the *Flavobacteria* (Figure 4B). The other phyla include *Proteobacteria* (16), *Actinobacteria* (2), and *Thermotogae* (1) (Figure 4A). Within the *Proteobacteria*, 14 affiliated with the *Epsilon* class (genus *Arcobacter*), and 2 with the *Gamma* class (Figure 4B). The TW 3–4.5 cm layer was dominated by the *Aquifiacae* (13 of 23 clones; Figures 4A,B), especially *Thermosulfidibacter spp*. (often with 100% confidence). The other phyla in this sample included *Actinobacteria*, *Planctomycetes*, *Proteobacteria*, *Thermodesulfobacteria*, and *Thermotogae* (Figure 4A). The deepest (9–10.5 cm) sediment sample at TW was dominated by the *Proteobacteria*, followed by *Bacteroidetes*, *Thermotogae*, and *Chloroflexi* (Figure 4A). Within the *Proteobacteria*, the *Epsilon* class (*Arcobacter spp*.; 18 of 27) dominated, followed by the *Beta* (3) and *Gamma* classes (2) (Figure 4B).

**Figure 5:**
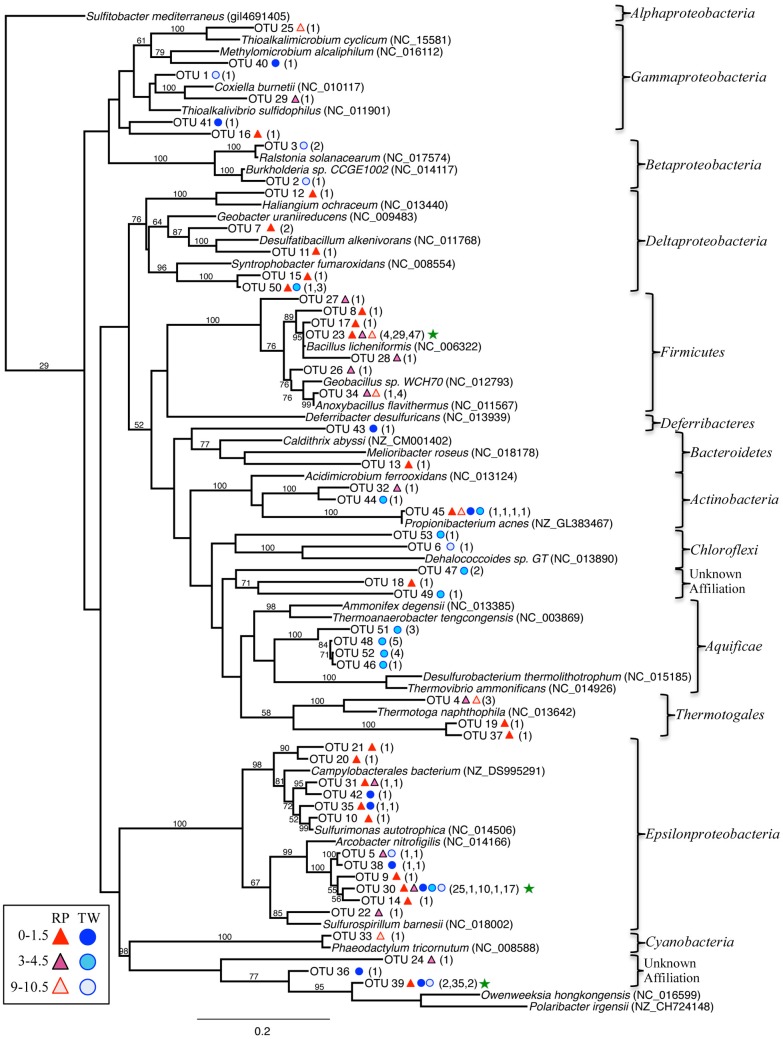
**Maximum-likelihood phylogenetic tree of closest relatives found for representatives from the 16S rRNA bacterial clone libraries**. OTUs are defined by a 97% similarity cutoff. Symbols next to the OTUs from this study represent the samples from which they were recovered, as indicated by the sample key at the bottom left. The number of clones belonging to each OTU per site is in parentheses. Bootstrap values of >50% are shown at nodes. *Sulfitobacter mediterraneus* was used as an outgroup. Green stars indicate the OTUs with the highest number of clones.

The bacterial 16S rRNA phylogenetic tree (Figure 5) includes the OTUs from our 16S rRNA clone libraries and some of their closest relatives, both isolates and other uncultured clones. Three OTUs represented >5 clones each, which are noted in the Figure 5 with a green star (173 total clones), and 50 OTUs represented ≤5 clones (78 total clones). OTU 23 represents 80 clones from the RP site (4 in the top layer, 29 in the middle, and 47 in the deepest layer). This OTU closely affiliates with the *Firmicutes* class (*Bacillus spp*.; Figure 5). OTU 30 (54 clones) affiliates with the *Epsilonproteobacteria* and was identified in all samples, except RP 9–10.5 cm. OTU 39 (39 clones) affiliated with *Bacteroidetes* and was identified almost exclusively in the TW 0–1.5 cm layer.

#### Archaeal affiliations

**Figure 6:**
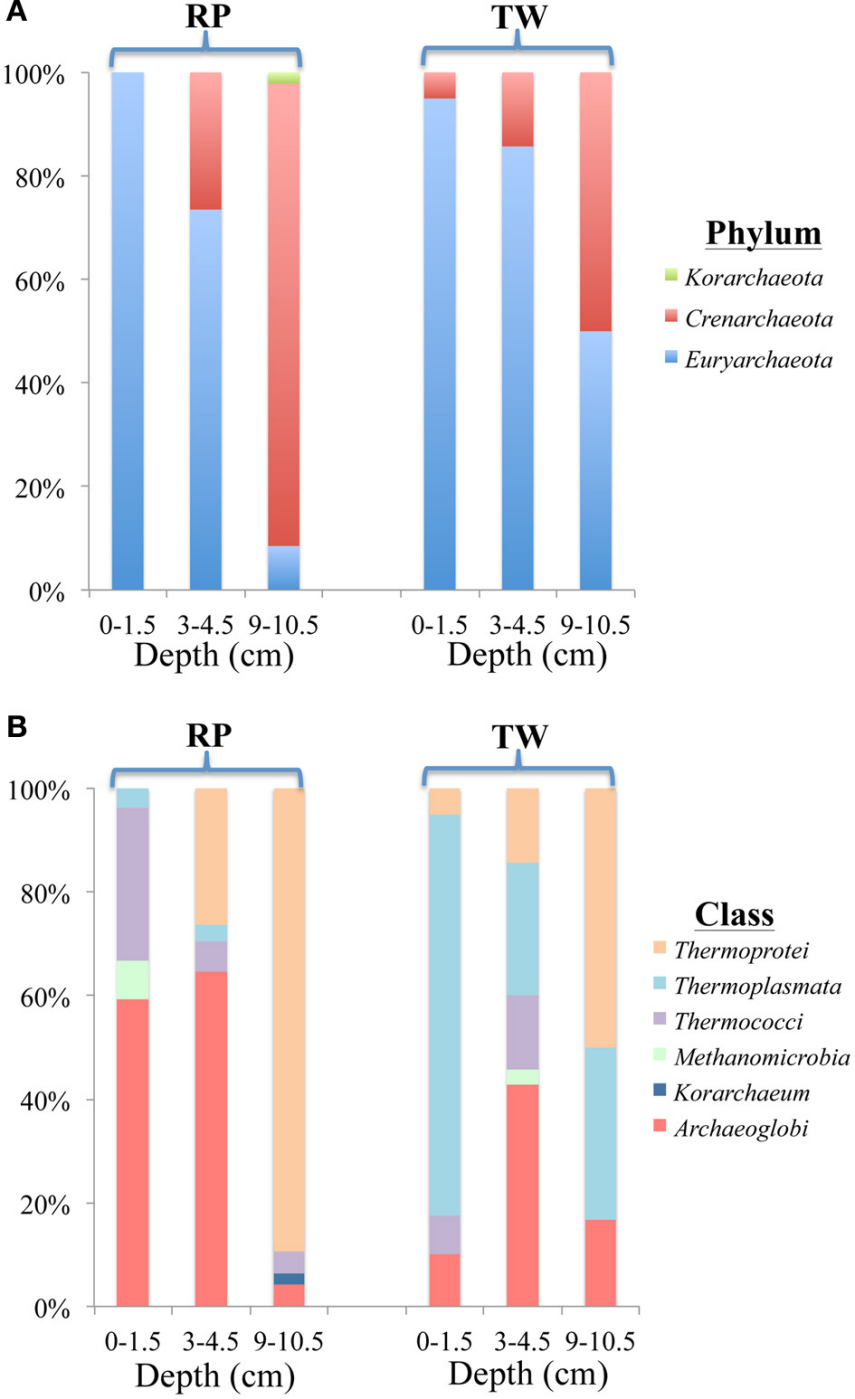
**Diversity and distribution of clones within archaeal phylum (A) and class (B) clone libraries for three depths of RP and TW sediment layers investigated in this study**. Each color represents the percentages of each taxon in the total respective clone library, as determined by screening of full length 16S rRNA gene sequences. Depths are 0–1.5, 3–4.5, and 9–10.5 cm, and indicated at the bottom of site designation.

The diversity and distribution of archaeal clones at the three depths of RP and TW sediments is shown in Figures 6A (phylum) and B (class). At both sites, *Euryarchaeota* dominated in the surface layers (Figure 6A). At RP, most of the clones affiliated with the *Archaeoglobi* (16/27; specifically the genus *Ferroglobus*) and *Thermococci* (8/27; specifically the genera *Palaeococcus* and *Thermococcus*) (Figure 6B). At TW, clones affiliating with *Thermoplasmata* (*Euryarchaeota*) dominated (31 of 40 clones; Figure 6B; specifically the genus *Thermogymnomonas*). Two clones affiliated with *Thermoprotei* (*Crenarchaota*). At both sites, with increasing depth, the phyla changed from predominantly *Euryarcharota* to more and mostly *Crenarchaeota*. The middle layer at RP (3–4.5 cm) was dominated by *Archaeoglobi* (*Euryarchaeota*), followed by *Thermoprotei* (*Crenarchaeota*; Figure 6B). The *Archaeoglobi* were again affiliated with genus *Ferroglobus*, while the *Thermoprotei* genera included *Staphylothermus*, *Pyrodictium*, and *Thermofilum*. The deepest RP site (9–10.5 cm) was dominated by *Thermoprotei* (*Crenarchaeota*; Figure 6B), with 42 of 47 clones affiliating with 5 genera including *Thermofilum* (25) and *Staphylothermus* (10). In the middle TW layer (3–4.5 cm), *Euryarchaeota* affiliated predominantly with *Archaeoglobi* (15; *Ferroglobus sp*.), and *Thermoplasmata* (9; *Thermogymnomonas sp*.), while the *Crenarchaeota* were entirely composed of *Thermoprotei* (5) (Figure 6B; specifically the genera *Hyperthermus* and *Thermofilum*). The TW 9–10.5 sequences affiliated with *Thermoprotei* (*Crenarchaeota*; 30 clones; genus *Thermofilum*), *Archaeoglobi* (*Euryarchaeota*; 11 clones; genus *Ferroglobus*) and *Thermoplasmata* (*Euryarchaeota*; 22 clones; genus *Thermogymnomonas*) (Figure 6B). Note, only one clone in all the libraries (RP 9–10.5) affiliated with the *Korarchaeota*.

**Figure 7:**
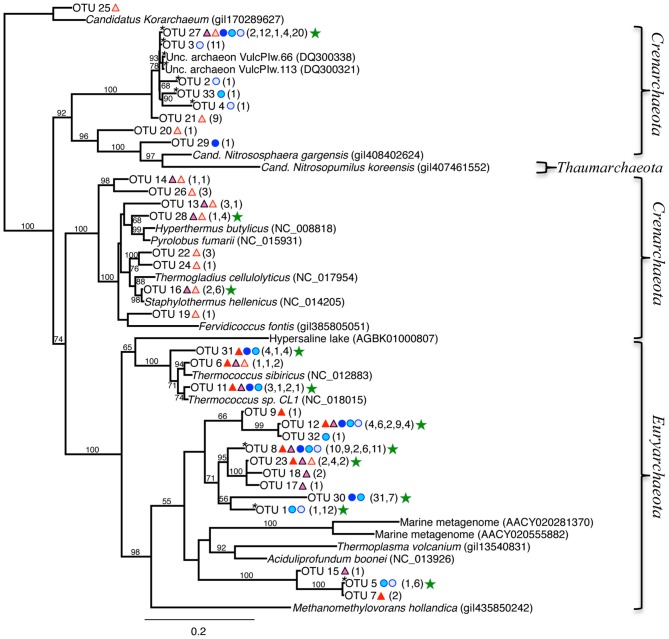
**Maximum-likelihood phylogenetic tree of closest relatives found for representatives from the 16S rRNA archaeal clone libraries**. OTUs are defined by a 97% similarity cutoff. Symbols next to the OTUs from this study represent the samples from which they were recovered, as indicated by the sample key in Figure 5. The number of clones belonging to each OTU per site is in parentheses. Bootstrap values of >50% are shown at nodes. *Methanomethylovorans hollandica* was used as an outgroup. Sequences containing inserts are noted with an “*.” Green stars indicate the OTUs with the highest number of clones.

The 16S rRNA archaeal phylogenetic tree (Figure 7) includes the OTUs from our 16S rRNA clone libraries and some of their closest relatives, other clones, and related isolates. Twelve OTUs represented >5 clones each, and are indicated by a green star in Figure 7 (212 total clones), and 20 OTUs represented ≤5 clones (37 total clones). Primary OTUs affiliated with *Thermoprotei* (*Crenarchaeota*), and *Thermococci* (*Euryarchaeota*; Figure 7). Many sequences were <90% similar to other archaeal representatives found in public databases and therefore do not cluster with any major group. A considerable fraction of our archaeal sequences (~20%) contained large ~26–80 bp “inserts,” possibly introns (Burggraf et al., 1993). The OTU sequences containing these inserts (OTUs 8, 1, 30, 5, 3, 27, 2, 33, and 4) passed comprehensive quality and chimera checks, and are therefore included in the phylogenetic tree, and indicated with an asterisk, “*” (Figure 7). BLAST hits indicate they are related to the Euryarchaeota (*Archaeoglobi* or *Thermoplasmata*), and *Crenarchaeota* (*Thermoprotaeles* or *Desulfurococcales*). Five of the top six OTUs with the highest number of clones contained these inserts (8, 1, 30, 3, and 27). OTU 27 (39 clones) was found in all samples except RP 0–1.5 (Figure 7), and is identifiable to *Thermoprotei* according to the RDP classification. Three OTUs (8, 30, and 1; 38, 38, and 13 clones, respectively) were distantly related to *Archaeoglobi* or *Thermoplasmata*. OTU 12 (25 clones) poorly affiliated with *Archaeoglobi* (38% ID), which results in it being separate from the other groups in the phylogenetic tree (Figure 7, top left).

#### Arsenic functional genes

**Figure 8:**
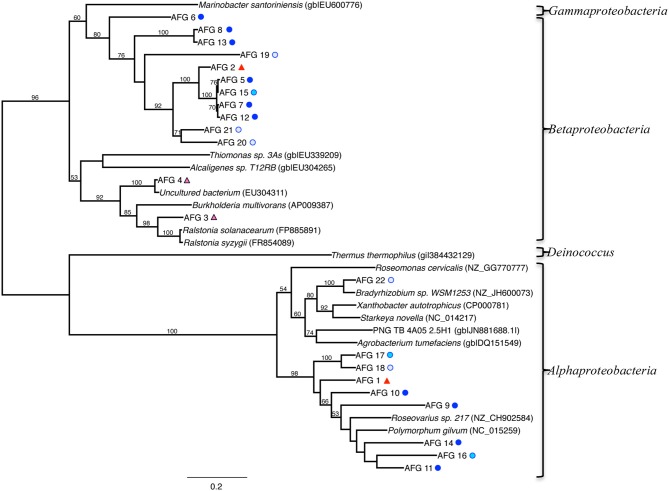
**Maximum-likelihood phylogenetic tree of closest relatives found for representatives from the *aioA*-like functional gene (AFG) clone libraries**. AFGs are defined by a 97% similarity cutoff. Symbols next to the OTUs from this study represent the samples from which they were recovered, as indicated by the sample key in Figure 5. Bootstrap values of >50% are shown at nodes. *Marinobacter santoriniensis* was used as an outgroup.

A phylogenetic tree of the *aioA*-like functional genes (AFGs) is shown in Figure 8, and includes sequences from our clone library, related isolates and other uncultured clones. Sequences affiliated with those detected in *Beta* (*Ralstonia* and *Burkholderia* genera), and *Alpha* classes of the *Proteobacteria* (*Polymorphum*, *Roseovarius* and *Bradyrhizobium* genera) cluster into two distinct lineages (Figure 8). Most of the AFGs associated with *Beta* class had no closely related cultured representative. A total of 105 clones across 22 taxa contained the AFG. Of these, 93 were from the TW site, with 57, 24, and 12 clones from the surface, middle, and deepest layers, respectively. At the RP site, the AFG was found only in the surface (5 clones) and middle (7 clones) layers.

## Discussion

### Microbial diversity

Our 16S rRNA gene sequence data indicate that bacterial and archaeal communities in the hydrothermally influenced sediments of Palaeochori Bay differ significantly when comparing the same depths of the RP high salinity and TW low salinity sites, although they have approximately the same temperature and pH (Figures 4–8, Table 1). Archaeal and bacterial class distribution between each site and vs. depth is shown in Table 4, and indicates that several classes were exclusive to either the RP or TW site. For example, comparing the 0–1.5 cm sediment depth, *Aquificae*, *Deferribacteres*, *Bacilli*, and *Deltaproteobacteria* were only present in the RP site, whereas *Sphingobacteria* and *Thermotogae* were only present in the TW sample (Table 4; Figures 4A,B). The same is true for the other sediment horizons, with distinct bacterial affiliations only occurring at one or the other site. Of the major bacterial groups, the *Firmicutes* were only present in the RP samples, while none were present in any of the TW sample depths. The 9–10.5 layer has zero overlapping bacterial classes between RP and TW sites (Table 4). *Betaproteobacteria* were identified in the TW sample from this depth, the only time this group was seen in any of the samples.

**Table 4:** **Bacterial and archaeal class distribution arranged by site and by depth**.

**Depth (cm)**	**Bacterial class**		**Archaeal class**	
	**RP**	**TW**	**RP**	**TW**
0–1.5	*Actinobacteria*	*Actinobacteria*	NP	*Thermoprotei*
	*Aquificae*	NP	*Archaeoglobi*	*Archaeoglobi*
	*Flavobacteria*	*Flavobacteria*	*Methanomicrobia*	NP
	*Deferribacteres*	NP	*Thermococci*	*Thermococci*
	*Bacilli*	NP	*Thermoplasmata*	*Thermoplasmata*
	NP^*^	*Sphingobacteria*		
	*Deltaproteobacteria*	NP		
	*Epsilonproteobacteria*	*Epsilonproteobacteria*		
	*Gammaproteobacteria*	*Gammaproteobacteria*		
	NP	*Thermotogae*		
3–4.5	*Actinobacteria*	*Actinobacteria*	*Thermoprotei*	*Thermoprotei*
	*Sphingobacteria*	NP	*Archaeoglobi*	*Archaeoglobi*
	*Bacilli*	NP	NP	*Methanomicrobia*
	NP	*Aquificae*	*Thermococci*	*Thermococci*
	NP	*Planctomycetacia*	*Thermoplasmata*	*Thermoplasmata*
	NP	*Deltaproteobacteria*		
	*Epsilonproteobacteria*	*Epsilonproteobacteria*		
	*Gammaproteobacteria*	NP		
	NP	*Thermodesulfobacteria*		
	NP	*Thermotogae*		
9–10.5	*Actinobacteria*	NP	*Thermoprotei*	*Thermoprotei*
	*Chloroflexi*	NP	*Archaeoglobi*	*Archaeoglobi*
	*Bacilli*	NP	NP	*Thermoplasmata*
	NP	*Flavobacteria*	*Thermococci*	NP
	NP	*Thermomicrobia*	*Candidatus Korarchaeum*	NP
	NP	*Betaproteobacteria*		
	NP	*Epsilonproteobacteria*		
	NP	*Gammaproteobacteria*		
	NP	*Thermotogae*		

* *NP, not present*.

Differences in the archaeal communities in surface samples are also quite distinct; e.g., *Archaeoglobi* is dominant at the RP site while *Thermoplasmata* was dominant at the TW site (Figures 6A,B). As was the case for the *Bacteria*, there exist some groups at RP, which were not found at the TW site, and vice versa (Table 4). For example, the RP surface sample did not contain *Thermoprotei*, a dominant archaeal group for the TW site. In the deepest layer, the RP site contained *Thermococci*, but no *Thermoplasmata* (Table 4). These results suggest that the major archaeal and bacterial groups in Palaeochori Bay are distinctly different in the high vs. low salinity sites.

### The role of phase separation

Subcritical phase separation is a ubiquitous process in hydrothermal systems, but its effect on microbial communities is rarely evaluated. Investigations of the Iheya North Field back-arc hydrothermal system south of Japan, suggested that subseafloor phase-separation and segregation may influence the supply of energy and carbon to vent-associated chemolithoautotrophs and related microbial communities (Nakagawa et al., 2005). When comparing microbial communities of gas-depleted “normal” fluids (i.e., those related to the remaining fluid phase after phase separation has taken place) to gas-enriched fluids (i.e., those which are related to the vapor phase; German and Von Damm, 2003), chemolithotrophs associated with gas-dependent energy metabolism, such as hydrogenotrophic methanogenesis, were more abundant. Another study investigated differences in microbial communities in phase separated hydrothermal fluids from the Yonaguni Knoll IV hydrothermal field in the southern Okinawa Trough (Nunoura and Takai, 2009). While fluids showed variability in the physical and chemical compositions (e.g., gas content), the microbial communities were relatively similar. There, chemolithotrophic hydrogen oxidation was the primary metabolism, and it was suggested that similarities in microbial communities could have been due to elevated concentrations of hydrogen in both types of fluids. Thus, microbial diversity may be influenced by a change in the concentration of potential electron donors as a result of phase separation (i.e., dissolved gases such as H_2_), not necessarily from salinity differences alone, although halotolerance cannot be ruled out. As we discuss below, metabolisms based on dissolved gases such as H_2_ are possible at each site investigated here, but do not seem to play a dominant role in influencing major groups.

Our geochemical results indicate that salinity and arsenic concentrations are among the most variable parameters when comparing RP with TW pore fluid compositions (Tables 1, 2; Figure 3). Free gas data indicate that O_2_, N_2_, He, H_2_, CO, and CH_4_ were only slightly higher for TW compared to RP (Table 2). Dissolved gas data suggest that no significant differences between sites exists for O_2_, CO, and H_2_ concentrations; the dissolved CH_4_ is slightly higher in the TW pore fluids (Table 2). For the redox sensitive species analyzed in this study, dissolved Fe^2+^, NO^−^_3_, and NO^−^_2_ were slightly higher, while NH_3_ was slightly lower, in fluids from the TW site (Table 1). Hydrogen sulfide concentrations can be very elevated (up to 3 mM) in high salinity fluids, but were essentially equal (~0.25 mM) in both high- and low-salinity hydrothermal fluids analyzed for this study (Druschel et al., unpublished data; Table 1). These geochemical parameters suggest that the phase separation process at Milos may create differences in concentrations of dissolved gases, as well as some other important redox couples, which could ultimately be contributing to the variability in microbial communities when comparing high- vs. low-salinity sites. However, we suggest that these differences are not significant, and therefore differences in microbial diversity may be influenced more by salinity and/or arsenic concentrations.

### Dominant bacterial metabolisms

The genus *Arcobacter*, which dominates the RP 0–1.5 cm sample, can grow at moderately thermophilic and halophilic conditions, utilizing nitrogen or sulfur, including sulfide oxidation (Wirsen, 2004; Donachie et al., 2005; Pati et al., 2010). For example, *A. nitrofigilis*, isolated from the roots of a salt marsh plant, can fix nitrogen (Pati et al., 2010). “*Candidatus* Arcobacter sulfidicus” has been shown to produce large white sulfur mats in hydrothermal environments (Wirsen, 2004), and related organisms may be responsible for the large white mats observed in Palaeochori Bay (Sievert et al., 1999). The white sulfur mats contain elemental sulfur, which may be the end product of sulfide oxidation. However, a comparison of our representative 16S rRNA gene sequence (OTU 30; Figure 5) against this and other *Arcobacter* species indicated only a 91–93% sequence similarity (BLAST comparison Max ID).

In the deeper RP sediments, *Firmicutes* dominate, in particular, *Bacillus sp*. This group is phenotypically highly diverse, and includes obligate aerobes, facultative anaerobes, chemotrophs, and heterotrophs, halotolerant members, and organisms that tolerate a wide range of temperatures (Graumann, 2012). Our representative sequence (OTU 23) is most closely related to *B. licheniformis* DSM 13 (Figure 5) which, along with many other members of the *Bacilli*, can reduce nitrate to N_2_ (Nakano and Zuber, 1998). Other investigations suggest that Mn(II) oxidation (Dick et al., 2006) and arsenate reduction may also be mediated by *Bacillus spp*. (Switzer et al., 1998). Four new strains of *Bacillus* were isolated from shallow-sea hydrothermal vents—two from Panarea and Vulcano Islands (Italy) (Maugeri et al., 2002; Spano et al., 2013), and two from the sediments in Palaeochori Bay (Milos) (Sievert et al., 2000a; Schlesner et al., 2001). The Milos isolates are *Halothiobacillus kellyi*, a mesophilic, obligately autotrophic, strictly aerobic, chemolithoautotrophic, sulfur-oxidizing bacterium (Sievert et al., 2000a), and *Filobacillus milensis*, a halophilic, faculatively anaerobic, endospore-forming heterotroph (Schlesner et al., 2001). In addition, 39 of 80 aerobic mesophiles isolated from Milos by Dando et al. (1998) are phenotypically similar to *Bacillus sp*.

The most abundant bacterial group in the surface sample at Twinkie (TW 0–1.5) was *Bacteroidetes*. However, the RDP classification confidence was quite low even at the class level (average 74%), suggesting many possibilities for representatives from this phylum. The representative OTU for these sequences is OTU 39, which had 35 out of 39 clones in the TW 0–1.5 cm sample. Due to the low confidence identification of this group, it is not affiliated with any other main group in the bacterial phylogentic tree (Figure 5, bottom left). BLAST results most closely identified with uncultured clones, making identification of primary metabolisms difficult. The phylum *Bacteroidetes* contains among the most abundant microbes in coastal marine waters (Alonso et al., 2007). Cultured representatives from this phylum are typically heterotrophs, non-thermophilic, and use complex organic substrates, either by aerobic respiration or fermentation (Kirchman, 2002). They can also be found in hydrothermal environments. For example, Kormas et al. (2006) detected *Bacteroidetes spp*. on a white smoker chimney at 9° N, East Pacific Rise. Recently, Sylvan et al. (2012) suggested that *Bacteroidetes* replaced “normal” microbial communities once smoker chimneys become inactive. Sievert et al. (2000b) and Nitzsche (2010) both identified *Bacteroidetes* as dominant members in the white areas from Milos sediments, but temperatures were in both cases quite lower than for our study (~20°C less). This suggests the possibility that *Bacteroidetes* activity is limited to lower temperature environments. Temperatures were comparable between the RP and TW sites (i.e., < ~40°C), but salinity was quite different, suggesting the lack of *Arcobacter* in place of *Bacteroidetes* at the TW site may also be influenced by halotolerance. However, as discussed, dissolved gases and other redox couple concentrations may play a role, as they are slightly elevated in the TW pore fluids.

The TW 3–4.5 site was dominated by *Thermosulfidibacter sp*., whose closest cultured representative is *Thermosulfidibacter takaii*, a thermophilic, sulfur-reducing chemolithoautotroph from a deep-sea hydrothermal vent at the Yonaguni Knoll IV, Southern Okinawa Trouph (Nunoura et al., 2008). Growth was observed within the temperature range of 55–78°C, and 0.5–4.5% NaCl. Temperatures at this sediment depth should be near 50°C, and salinity should be slightly lower than seawater (Table 1; Figure 3).

The TW 9–10.5 was dominated by *Arcobacter spp*. (*Epsilonproteobacteria*; 18 out of 27 clones). As discussed, the group could be conducting nitrate reduction or sulfide oxidation. Comparing the OTU representative sequence (OTU 5) from TW 9–10.5 to the 16S rRNA gene sequence from *A. nitrofigilus* and “*Ca*. A. *sulfidicus*” indicates BLAST max ID of 93% and 93%, respectively. Indeed, the representative OTUs from RP 0–1.5 (OTU 14) and TW 9–10.5 (OTU 5) were only 96% max ID when compared to each other. This suggests that the *Arcobacter* species in both the RP 0–15 and TW 9–10.5 samples are only distantly related, and it is probable that they are different species or genera with different environmental tolerances.

These data indicate that bacterial communities are stratified by depth, and that sulfur- and nitrogen-based metabolisms may dominate at the RP site, whereas phototrophy, sulfur, and or nitrogen-based metabolisms may be dominant at the TW site. Temperature may be the dominant parameter which influences microbial community structure as a function of depth at both RP and TW sites, as vertical gradients are more pronounced for this parameter compared to any other (Table 1; Figure 4). Sunlight may also play a role, particularly for the surface TW site where *Bacteroidetes* is present.

### Dominant archaeal metabolisms

The dominant archaeal genera for RP 0–1.5 were *Ferroglobus* (*Archaeoglobi*) and *Palaeococcus*/*Thermococcus* (*Thermococci*). These groups become replaced mostly by the *Thermoprotei* (*Staphylothermus* and *Thermophilum*) at depth. However, poor RDP classification confidence makes identification beyond the phylum level difficult. Further complicating matters, the OTU chosen to represent the sequences associated with the *Archaeoglobi* (OTU 8) contained a 44 bp insert. BLAST analysis of OTU 8 returned no cultured representatives, and identified most closely with uncultured clones from the Logatchev hydrothermal field (LHF) and terrestrial hydrothermal vents on Ambitle Island, PNG. Removing the insert from OTU 8 and conducting a BLAST analysis for the remaining sequence indicates *Aciduliprofundum spp*. as the closest cultured match (100% query coverage; 86% max ID). This organism belongs to the “Deep-sea Hydrothermal Vent Euryarchaeota 2” (DHVE2) lineage (Takai and Horikoshi, 1999), which are widespread at deep-sea hydrothermal vents (Reysenbach et al., 2006; Flores et al., 2012). Unfortunately, relatively little is known about their distribution and phylogenetic diversity (Flores et al., 2012). The only described species from the *Aciduliprofundum* genus is *A. boonei* T469^T^, which is an obligate thermoacidophilic sulfur- or iron-reducing heterotroph capable of growing from pH 3.3–5.8 and between 55 and 75°C (Reysenbach et al., 2006). This organism is included in the phylogenetic tree, but does not affiliate closely with our sequences (Figure 7).

The second most abundant archaeal group in the RP 0–1.5 sample was *Thermococci*, consisting of both *Thermococcus spp*. and *Palaeococcus spp*., with ~100% RDP confidence. Our OTUs for this group (31, 6, and 11) did not contain insertions, and identified most closely with *Thermococcus spp*. (Figure 7). There are numerous species related to this genus; most are considered extreme thermophiles, typically identified as organotrophic anaerobes which grow above 70°C, sometimes requiring elemental S, producing H_2_S and CO_2_, and have the ability to use H^+^ (Arab et al., 2000).

The RP 9–10.5 sample was dominated by the *Crenarchaeota*–*Thermoprotei*, was composed of several genera, which were dominated by *Thermofilum spp.*, although mostly with very poor confidence, followed by *Staphylothermus spp*., often with 100% confidence, and several others. Our OTUs chosen to represent the *Thermofilum spp*. identified by the RDP classification (21, 26, and 27) most closely affiliated with *Thermoprotei* (Figure 7). However, OTU 26 is within a slightly different cluster, and had much higher RDP % confidence than the other two with *Thermofilum spp*. BLAST analysis of this OTU most closely matched with *Thermofilum pendens*, a thermophilic, anaerobic sulfur-respirer. OTU 27 contained an insert, and BLAST analysis indicated the closest representative was the uncultured clone VulcPlw.113, from the shallow-sea hydrothermal vents off Vulcano, Italy. The sequence from this clone, as noted, also contains an insert. Removing the insert from OTU 27 and conducting a BLAST analysis indicates it does not affiliate with any cultured representatives, and is most closely related to various uncultured *Archaea* from the Mariana Trough, East Pacific Rise, and the shallow-sea vents off Ambitle Island, Papua New Guinea (Meyer-Dombard et al., 2011).

The TW 0–1.5 was by far dominated by the *Thermoplasmata* class, *Thermogymnomonas* genus. This genus has one heterotrophic species, *T. acidicola*, and can grow at temperatures in the range 38–68°C (optimally at 60°C), at pH 1.8–4.0 (optimally at around pH 3.0), and is obligately aerobic and heterotrophic, requiring yeast extract for growth (Itoh et al., 2007). These clones are represented by OTU 30, which according to RDP, poorly affiliated with *Thermoprotei*. This OTU contained an insert, and BLAST analysis indicated affiliation to uncultured clones VulcPlw.76, and those from terrestrial vents from Papua New Guinea. The closest cultured representative was *Aciduliprofundum sp*. MAR08-237A. BLAST analysis of this sequence with the insert removed indicates similar results.

In the deeper 9–10.5 sample for TW, dominant members transition to *Thermoprotei* (mostly *Thermofilum spp*.; OTUs 2 and 3), although abundant *Thermoplasmata* (*Thermogymnomonas spp*.; OTUs 1 and 5) were also present. Metabolisms for these organisms have been discussed previously. Each of these OTUs contained inserts. OTUs 2 and 3 identified most closely with VulcPLw.76 uncultured clone and previously obtained clone sequences from Milos, with no affiliation to a cultured representative. OTUs 1 and 5 identified with the isolate *A. boonei*. OTU 4 in this sample identified with *Stetteria sp*., from the *Thermoprotei*–*Desulfurococcaceae* family, by the RDP classification. BLAST analysis of this sequenced indicates affiliation with the previously described uncultured clones from the shallow-sea vents off Vulcano and Milos.

These results indicate that archaeal metabolisms in both surface and deeper sediments are dominated by sulfur oxidizers, can be heterotrophic, and may also have the ability to conduct Fe oxidation. These poorly classified groups, particularly those which contain an insert, could represent new genera or new higher order taxa.

Although we cannot say for sure these extra nucleotide sequence insertions are introns, we believe the extra nucleotides in our sequences are real for three main reasons: (1) The quality scores for most of these sequences were very high. (2) Multiple chimera checks suggested they are not chimeras (see Materials and Methods section), (3) They are found not only in our dataset, but also in several other datasets from Milos [Brehmer, 2000 (unpublished data, NCBI database); Nitzsche, 2010], and the near-shore hydrothermal vent system of Vulcano (Italy) (Rogers and Amend, 2005). The other datasets were obtained from completely different laboratories using different methods, making it highly unlikely that there is an error. All of the other introns found in this study, when blasted independently after exons were removed, consistently returned affiliations with clone sequences from these previously reported groups as the closest representatives. An intron is an additional piece of nucleotide sequence found in the genes of most organisms and many viruses, and can be located in a wide range of genes, including those that generate proteins, rRNA, and tRNA (Itoh et al., 2003). Introns in the 16S rRNA gene of *Archaea* are rare, and primarily restricted to the *Crenarchaeota*, within the families *Thermoproteaceae* and *Desulfurococcales* (Morinaga et al., 2002; Itoh et al., 2003). Although outside the current scope of this paper, it is important to note that if the extra nucleotide sequence contained within our archaeal sequences for OTU 8, as well as OTUs 1, 30, and 5, are in fact introns, it is a significant finding as introns contained within the 16S rRNA gene sequences of *Euryarchaeota* have not, to our knowledge, been reported.

### Arsenic functional gene survey

Elevated arsenic concentrations may clearly influence microbial community structure and function, since the concentrations encountered can be toxic and may also provide additional redox couples for microbial metabolism (e.g., arsenotrophy). For example, in reducing hydrothermal fluids, arsenic exists most commonly as arsenite, with a +3 oxidation state (AsIII, H_2_AsO^−^_3_). However, in surface seawater and other oxidizing environments, arsenic is dominantly in the +5 oxidation state (arsenate, AsV, HAsO^2−^_4_). Thus, microorganisms can play an important role in the arsenic cycle as they catalyze the transformation of different arsenic redox species (Handley et al., 2009; Akerman et al., 2011; Meyer-Dombard et al., 2011, 2013). The three main types of arsenotrophs thus far known to occur are heterotrophic arsenite oxidizers (HAOs), chemotrophic arsenite oxidizers (CAOs), and dissimilatory arsenate-reducing prokaryotes (DARPs; Oremland and Stolz, 2003). Genes involved in these arsenic transformations include detoxification via the ArsC system, and energy gain via the arsenate respiratory reductase (two subunits, ArrA and ArrB) or the arsenite oxidase (two subunits, AioA and AioB, formerly referred to as AroA and/or AoxB; Oremland and Stolz, 2003; Zargar et al., 2010). Recently, an alternative AsIII-oxidation gene was discovered (arxA-type; Zargar et al., 2010). To date, most studies of As microbial cycling have focused on various naturally or anthropogenically impacted environments, primarily located in terrestrial (non-marine) settings. Next to nothing is known about As cycling in coastal marine environments.

At the two sites investigated here, analysis of the AFGs revealed affiliation with only two classes within the *Proteobacteria* phylum, although we must keep in mind that lateral gene transfer may have occurred (Figure 8). The *Alphaproteobacteria* containing the arsenite oxidase gene were predominantly affiliated with the genera *Polymorphum, Bradyrhizobium* and *Roseovarious*, while the *Betaproteobacteria* were affiliated with the *Burkholderia* or *Ralstonia* genera. The primers used for screening of *aioA*-like genes were based on a range of groups, and therefore should be broadly applicable (i.e., should not be specific to one or two groups). For example, primer design relied on the sequence alignment of 16 arsenite oxidase amino acid sequences consisting of 5 *Alpha*-, 4 *Beta*-, 3 *Gammaproteobacteria*, 2 *Deinococci*, and one *Chloroflexi* (Zargar et al., 2012). Thus, there could be a bias toward *Alpha*- and *Betaproteobacteria*, but many published arsenite oxidizers are in these groups (e.g., the most updated phylogenetic tree for arsenotrophs has many of the *aioA*-like gene containing microbes as *Alpha-* and *Betaproteobacteria*). There are some newer isolates being reported within the *Gammaproteobacteria*, and various genome sequencing projects are also starting to show the presence of *aioA*-like genes (Handley et al., 2009; Cavalca et al., 2013). The primers used here were the best available at the time of their design.

AFG distribution was primarily associated with the TW samples, which is somewhat expected given the higher concentrations of arsenic at this site (Figure 8). Phylogenetic analysis revealed that both the *Beta*- and *Alphaproteobacteria* relationships can be further subdivided into two distinct lineages (Figure 8). While some of the *Betaproteobacteria* were most closely related to *Ralstonia* and *Burkholderia* genera (AFGs 3 and 4), nearly all of the other AFGs associated with *Betaproteobacteria* had no closely related cultured representative (Figure 8). Four main clusters exist for these *Betaproteobacteria*. They consist of (1) AFG 6, (2) AFGs 2, 5, 15, 7, 12, 20, and 21, (3) AFG 19, and (4) AFGs 8 and 13. BLAST analysis of these AFGs indicate they are associated with either *Beta*- or *Alphaproteobacteria*, mostly *Burkholderiales*, but cannot be constrained beyond the *Proteobacteria* phylum. The *Alphaproteobacteria* were most closely related either to *Polymorphum*, *Roseovarius*, or *Bradyrhizobium spp*. AFG 11 was closest to *Polymorphum gilvum* in the phylogenetic analysis (Figure 8). Only *Burkholderiales* occurred in the low salinity 9–10.5 cm depth in the 16S rRNA gene clone libraries. BLAST analysis of these AFG sequences indicates similarity to *aioA*-like genes from various terrestrial arsenic-rich environments.

These species are only distantly related, and therefore discussion of their arsenic oxidation strategy is limited. For example, *Polymorphum gilvum* was isolated from a crude oil-polluted saline soil in Shengli Oilfield, China, and was able to use the crude oil as the sole carbon source, and is not known to conduct arsenic metabolism, although its genome contains genes for both arsenite oxidation and arsenate reduction (detoxification) (Li et al., 2011). All of the related groups in the AFG analysis can be confirmed to contain an arsenite oxidase, but none of them have been shown to oxidize or reduce arsenic for detoxification or for their metabolism.

Overlap in AFG and 16S rRNA gene sequence data occurred only in the low salinity TW 9–10.5 sediments. For example, two OTUs (2 and 3) from the bacterial 16S rRNA data set were identified most closely with the *Betaproteobacteria*, specifically *Ralstonia sp*. and *Burkholderia sp*. This suggests arsenotrophy may be an important metabolism in deep, low-salinity, As-rich environments similar to those found in the TW 9–10.5 layer.

Other molecular investigations of the genes responsible for As redox transformations have revealed intriguing evidence for As microbial transformations in marine hydrothermal environments. For example, Nitzsche (2010) used arsenic primers for *aioA*-like gene amplification of 0–6 cm pooled sediments from Milos, and obtained similar results to our data (i.e., *Alpha-* and *Betaproteobacteria*). At a marine shallow-water hydrothermal vent off Ambitle Island, Papua New Guinea, microorganisms may be key in the oxidation of As(III) in areas immediately surrounding the vent source (Akerman, 2009; Meyer-Dombard et al., 2011, 2013). Using PCR primers designed to amplify arsenic functional genes, biofilm communities at the site were shown to possess the genetic capacity for the oxidation of As(III). The *aroA*-like (now known as *aioA*-like) clone sequences analyzed belonged to relatives of *Alpha*- and *Betaproteobacteria*, *Thermus*, and *Pyrobaculum*, but were only 71–80% similar to these sequences available in GenBank, suggesting that many of the Ambitle Island As-functional genes are unique. Thus, in marine environments, arsenite oxidation may be limited to the *Alpha-* and *Betaproteobacteria*, but much more research is necessary, particularly cultivation and molecular-based (next generation metagenomic) approaches focusing on arsenotrophy.

## Conclusions and outlook

Our results indicate that differences in archaeal and bacterial community structure exist at the site as a function of not only depth, but also when comparing high vs. low salinity environments with similar redox potential. A wide range of metabolisms seem possible, with affiliated members likely involved in the nitrogen or sulfur cycle. However, it should be noted that rare members of the community can also be very active and function as “keystone species,” yet not be detected in clone libraries. Differences in bacterial and archaeal communities between the RP high-salinity and TW low-salinity sites suggest geochemical signatures resulting from phase separation may be a controlling factor in hydrothermal vent microbial ecology (e.g., salinity, arsenic). Our screening of AFGs responsible for arsenite oxidation indicate that both the *Alpha-* and *Betaproteobacteria* may be responsible for arsenite oxidation in sediments from both sites, although the most abundant clusters are from the higher-arsenic TW site. These results are similar to previously reported AFG screening in marine environments, suggesting that *Alpha-* and *Betaproteobacteria* are the key players in marine hydrothermal arsenic metabolism. Since these groups are only distantly related to known arsenite oxidizers, novel genera may be present at the site; much more research is necessary including targeted cultivation of arsenotrophs. These results also provide incentive for metagenomic investigation of these sites to identify potential novel shallow vent species, and confirm arsenite metabolism is present primarily in the *Alpha-* and *Betaproteobacteria*. Affiliations from the arsenic gene screening were often not reflected in the 16S rRNA results, suggesting that arsenite may only be an important electron donor in the deeper, low salinity, high arsenic sediments. However, this could also be the result of a lack of sufficient database representation, relatively shallow sequencing depth or potential bias in the AFG primers. Finally, many of the archaeal 16S rRNA sequences contained putative introns, including members of *Euryarchaeota*, which has not been reported previously.

### Conflict of interest statement

The authors declare that the research was conducted in the absence of any commercial or financial relationships that could be construed as a potential conflict of interest.

## References

[B1] Akerman N. (2009). Microbial diversity and geochemical energy sources of Tutum Bay, Ambitle Island, Papua New Guinea, an arsenic-rich shallow-sea hydrothermal system. Ph.D. dissertation, Washington University in St. Louis.

[B2] Akerman N. Price R. Pichler T. Amend J. P. (2011). Energy sources for chemlithotrophs in an arsenic- and iron-rich shallow-sea hydrothermal system. Geobiology 9, 436–445 21884364 10.1111/j.1472-4669.2011.00291.x

[B3] Alonso C. Warnecke F. Amann R. Pernthaler J. (2007). High local and global diversity of *Flavobacteria* in marine plankton. Environ. Microbiol. 9, 1253–1266 10.1111/j.1462-2920.2007.01244.x 17472638

[B4] Amend J. P. Rogers K. L. Shock E. L. Gurrieri S. Inguaggiato S. (2003). Energetics of chemolithoautotrophy in the hydrothermal system of Vulcano Island, southern Italy. Geobiology 1, 37–58 10.1046/j.1472-4669.2003.00006.x

[B5] Arab H. Voelker H. Thomm M. (2000). *Thermococcus aegaeicus* sp. nov. and *Staphylothermus hellenicus* sp. nov., two novel hyperthermophilic archaea isolated from geothermally heated vents off Palaeochori Bay, Milos, Greece. Int. J. Syst. Evol. Microbiol. 50, 2101–2108 10.1099/00207713-50-6-2101 11155985

[B6] Bayraktarov E. Price R. E. Ferdelman T. G. Finster K. (2013). The pH and pCO2 dependence of sulfate reduction in shallow-sea hydrothermal CO2-venting sediments (Milos Island, Greece). Front. Microbiol. 4, 1–10 10.3389/fmicb.2013.00111 23658555 PMC3647119

[B8] Brinkhoff T. Sievert S. Kuever J. Muyzer G. (1999). Distribution and diversity of sulfur-oxidizing *Thiomicrospira spp*. at a shallow-water hydrothermal vent in the Aegean Sea (Milos, Greece). Appl. Environ. Microbiol. 65, 3843–3849 10473384 10.1128/aem.65.9.3843-3849.1999 PMC99709

[B9] Burggraf S. Larsen N. Woese C. R. Stetter K. (1993). An intron within the 16S ribosomal RNA gene of the archaeon *Pyrobaculum aerophilum*. Proc. Natl. Acad. Sci. U.S.A. 90, 2547–2550 10.1073/pnas.90.6.2547 8460170 PMC46125

[B10] Cavalca L. Corsini A. Zaccheo P. Andreoni V. Muyzer G. (2013). Microbial transformations of arsenic: perspectives for biological removal of arsenic from water. Future Microbiol. 8, 753–768 10.2217/fmb.13.38 23586329

[B11] Cox M. E. Launay J. Paris J. P. (1982). Geochemistry of low temperature geothermal systems in New Caledonia, in Pacific Geothermal Conference, (Auckland: University of Auckland), 453–459

[B12] Dando P. R. Thomm M. Arab H. Brehmer M. Hooper L. E. Jochimsen B. (1998). Microbiology of shallow hydrothermal sites off Palaeochori Bay, Milos (Hellenic Volcanic Arc). Cah. Biol. Mar. 39, 369–372

[B13] Dando P. R. Hughes J. A. Leahy Y. Niven S. J. Taylor L. J. Smith C. (1995). Gas venting rates from the submarine hydrothermal areas around the island of Milos, Hellenic volcanic arc. Cont. Shelf Res. 15, 913–929 10.1016/0278-4343(95)80002-U

[B14] Dando P. R. Stüben D. Varnavas S. P. (1999). Hydrothermalism in the Mediterranean Sea. Prog. Oceanogr. 44, 333–367 10.1016/S0079-6611(99)00032-4

[B15] Dick G. J. Lee Y. E. Tebo B. M. (2006). Manganese(II)-oxidizing *Bacillus* spores in Guaymas Basin hydrothermal sediments and plumes. Appl. Environ. Microbiol. 72, 3184–3190 10.1128/AEM.72.5.3184-3190.2006 16672456 PMC1472353

[B16] Donachie S. P. Bowman J. P. On S. L. W. Alam M. (2005). *Arcobacter halophilus* sp. nov., the first obligate halophile in the genus *Arcobacter*. Int. J. Syst. Evol. Microbiol. 55, 1271–1277 10.1099/ijs.0.63581-0 15879267

[B17] Flores G. E. Campbell J. H. Kirshtein J. D. Meneghin J. Podar M. Steinberg J. I. (2011). Microbial community structure of hydrothermal deposits from geochemically different vent fields along the Mid-Atlantic Ridge. Environ. Microbiol. 13, 2158–2171 10.1111/j.1462-2920.2011.02463.x 21418499

[B18] Flores G. E. Wagner I. D. Liu Y. Reysenbach A. L. (2012). Distribution, abundance, and diversity patterns of the thermoacidophilic “deep-sea hydrothermal vent euryarchaeota 2”. Front. Microbiol. 3:47 10.3389/fmicb.2012.00047 22363325 PMC3282477

[B19] Forrest M. J. Ledesma-Vazquez J. Ussler I, W. Kulongoski J. T. Hilton D. R. Greene H. G. (2005). Gas geochemistry of a shallow submarine hydrothermal vent associated with the El Requeson fault zone, Bahia Concepcion, Baja California Sur, Mexico. Chem. Geol. 224, 82–95 10.1016/j.chemgeo.2005.07.015

[B20] German C. R. Von Damm K. L. (2003). Hydrothermal processes, in Treatise on Geochemistry, eds Holland H. D. Turekian K. K. (Amsterdam: Elsevier), 145–180

[B21] Graumann P. (2012). Bacillus: Cellular and Molecular Biology. (Norfolk: Caister Academic Press).

[B22] Guindon S. Gascuel O. (2003). A simple, fast, and accurate algorithm to estimate large phylogenies by maximum likelihood. Syst. Biol. 52, 696–704 10.1080/10635150390235520 14530136

[B23] Handley K. M. Hery M. Lloyd J. R. (2009). Redox cycling of arsenic by the hydrothermal marine bacterium *Marinobacter santoriniensis*. Environ. Microbiol. 11, 1601–1611 10.1111/j.1462-2920.2009.01890.x 19226300

[B24] Huber T. Faulkner G. Hugenholtz P. (2004). Bellerophon; a program to detect chimeric sequences in multiple sequence alignments. Bioinformatics 20, 2317–2319 10.1093/bioinformatics/bth226 15073015

[B25] Italiano F. Bonfanti P. Ditta M. Petrini R. Slejko F. (2009). Helium and carbon isotopes in the dissolved gases of Friuli Region (NE Italy): geochemical evidence of CO2 production and degassing over a seismically active area. Chem. Geol. 266, 76–85 10.1016/j.chemgeo.2009.05.022

[B26] Italiano F. Nuccio F. (1991). Geochemical investigations of submarine volcanic exhalations to the east of Panarea, Aeolian Islands, Italy. J. Volcanol. Geother. Res. 46, 125–141 10.1016/0377-0273(91)90079-F

[B27] Itoh T. Nomura N. Sako Y. (2003). Distribution of 16S rRNA introns among the family Thermoproteaceae and their evolutionary implications. Extremophiles 7, 229–233 12768454 10.1007/s00792-003-0314-y

[B28] Itoh T. Yoshikawa N. Takashina T. (2007). *Thermogymnomonas acidicola* gen. nov., sp. nov., a novel thermoacidophilic, cell wall-less archaeon in the order Thermoplasmatales, isolated from a solfataric soil in Hakone, Japan. Int. J. Syst. Evol. Microbiol. 57, 2557–2561 10.1099/ijs.0.65203-0 17978217

[B29] Jochimsen B. Peinemann-Simon S. Volker H. Stuben D. Botz R. Stoffers P. (1997). *Stetteria hydrogenophila* gen. nov. and sp. nov., a novel mixotrophic sulfur-dependent *crenarchaeote* isolated from Milos, Greece. Extremophiles 1, 67–73 10.1007/s007920050016 9680304

[B30] Kirchman D. L. (2002). The ecology of *Cytophata-Flavobacteria* in aquatic environments. FEMS Microbiol. Ecol. 39, 91–100 10.1016/S0168-6496(01)00206-9 19709188

[B31] Kormas K. A. Tivey M. K. Von Damm K. L. Teske A. (2006). Bacterial and archaeal phylotypes associated with distinct mineralogical layers of a white smoker spire from a deep-sea hydrothermal vent site (9°N, East Pacific Rise). Environ. Microbiol. 8, 909–920 10.1111/j.1462-2920.2005.00978.x 16623747

[B32] Koski R. A. Pichler T. Foster A. L. (2001). The fate of arsenic in submarine hydrothermal environments: a summary of recent data, in USGS Workshop on Arsenic in the Environment (Denver, CO).

[B33] Lane D. J. (1991). 16S/23S rRNA sequencing, in Nucleic Acid Techniques in Bacterial Systematics, eds Stackebrandt E. Goodfellow M. (Chichester: John Wiley and Sons), 115–175

[B34] Li S.-G. Tang Y.-Q. Nie Y. Cai M. Wu X.-L. (2011). Complete genome sequence of *Polymorphum gilvum* SL003B-26A1T, a crude oil-degrading bacterium from oil polluted saline soil. J. Bacteriol. 193, 2894–2895 10.1128/JB.00333-11 21478361 PMC3133105

[B36] Maugeri T. L. Gugliandolo C. Caccamo D. Panico A. Lama L. Gambacorta A. (2002). A halophilic thermotolerant *Bacillus* isolated from a marine hot spring able to produce a new exopolysaccharide. Biotechnol. Lett. 24, 515–519 10.1023/A:1014891431233

[B37] McCarthy K. T. Pichler T. Price R. E. (2005). Geochemistry of Champagne Hot Springs shallow hydrothermal vent field and associated sediments, Dominica, Lesser Antilles. Chem. Geol. 224, 55–68 10.1016/j.chemgeo.2005.07.014

[B38] Meyer-Dombard D. Amend J. P. Osburn M. R. (2013). Microbial diversity and potential for arsenic and iron biogeochemical cycling at an arsenic-rich, shallow-sea hydrothermal vent (Tutum Bay, Papua New Guinea). Chem. Geol. 348, 37–47 10.1016/j.chemgeo.2012.02.02421884364

[B39] Meyer-Dombard D. R. Price R. E. Pichler T. Amend J. P. (2011). Prokaryotic populations in arsenic-rich shallow-sea hydrothermal sediments of Ambitle Island, Papua New Guinea. Geomicrobiol. J. 29, 1–17 21884364

[B40] Morinaga Y. Nomura N. Sako Y. (2002). Population dynamics of archaeal mobile introns in natural environments: a shrewd invasion strategy of the latent parasitic DNA. Microbes Environ. 17, 153–163 10.1264/jsme2.17.153

[B41] Nakagawa S. Takai K. Inagaki F. Chiba H. Ishibashi J.-I. Kataoka S. (2005). Variability in microbial community and venting chemistry in a sediment-hosted backarc hydrothermal system: impacts of subseafloor phase-separation. FEMS Microbiol. Ecol. 54, 141–155 10.1016/j.femsec.2005.03.007 16329980

[B42] Nakano M. M. Zuber P. (1998). Anaerobic growth of a “strict aerobe” (*Bacillus subtilis*). Annu. Rev. Microbiol. 52, 165–190 10.1146/annurev.micro.52.1.165 9891797

[B43] Nitzsche K. (2010). Microbial Diversity of Hydrothermally Influenced Arsenic-Rich Sediments off the Coast of Milos Island, Greece. Diploma thesis, TU Bergakademie, Freiberg, Germany.

[B44] Nunoura T. Oida H. Miyazaki M. Suzuki Y. (2008). *Thermosulfidibacter takaii* gen. nov., sp. nov., a thermophilic, hydrogen-oxidizing, sulfur-reducing chemolithoautotroph isolated from a deep-sea hydrothermal field in the Southern Okinawa Trough. Int. J. Syst. Evol. Microbiol. 58, 659–665 10.1099/ijs.0.64615-0 18319474

[B45] Nunoura T. Takai K. (2009). Comparison of microbial communities associated with phase-separation-induced hydrothermal fluids at the Yonaguni Knoll IV hydrothermal field, the Southern Okinawa Trough. FEMS Microbiol. Ecol. 67, 351–370 10.1111/j.1574-6941.2008.00636.x 19159423

[B46] Oremland R. S. Stolz J. F. (2003). The ecology of arsenic. Science 300, 939–944 10.1126/science.1081903 12738852

[B47] Pati A. Gronow S. Lapidus A. Copeland A. Glavina Del Rio T. Nolan M. (2010). Complete genome sequence of *Arcobacter nitrofigilis* type strain (CI). Stand. Genomic. Sci. 2, 300–308 10.4056/sigs.912121 21304714 PMC3035288

[B48] Pichler T. (2005). Stable and radiogenic isotopes as tracers for the origin, mixing and subsurface history of fluids in submarine shallow-water hydrothermal systems. J. Volcanol. Geother. Res. 139, 211–226 10.1016/j.jvolgeores.2004.08.007

[B49] Pichler T. Amend J. P. Garey J. Hallock P. Hsia N. P. Karlen D. J. (2006). A natural laboratory to study arsenic geobiocomplexity. EOS 87, 221–225 10.1029/2006EO230002

[B50] Pichler T. Giggenbach W. F. McInnes B. I. A. Duck B. (1999). Fe-sulfide formation due to seawater-gas-sediment interaction in a shallow-water hydrothermal system, Lihir Island, Papua New Guinea. Econ. Geol. 94, 281–288 10.2113/gsecongeo.94.2.281

[B51] Pokrovski G. S. Zakirov I. V. Roux J. Testemale D. Hazemann J. L. Bychkov A. (2002). Experimental study of arsenic speciation in vapor phase to 500°C: implications for As transport and fractionation in low-density crustal fluids and volcanic gases. Geochim. Cosmochim. Acta 66, 3453–3480 10.1016/S0016-7037(02)00946-8

[B52] Polz M. F. Cavanaugh C. M. (1995). Dominance of one bacterial phylotype at a Mid-Atlantic ridge hydrothermal vent site. Proc. Natl. Acad. Sci. U.S.A. 92, 7232–7236 10.1073/pnas.92.16.7232 7543678 PMC41313

[B53] Price R. E. Amend J. P. Pichler T. (2007). Enhanced geochemical gradients in a marine shallow-water hydrothermal system: unusual arsenic speciation in horizontal and vertical pore water profiles. Appl. Geochem. 22, 2595–2605 10.1016/j.apgeochem.2007.06.010

[B54] Price R. E. Savov I. Planer-Friedrich B. Bühring S. Amend J. P. Pichler T. (2012). Processes influencing extreme As enrichment in shallow-sea hydrothermal fluids of Milos Island, Greece. Chem. Geol. 348, 15–26 10.1016/j.chemgeo.2012.06.007

[B56] Reysenbach A. L. Liu Y. Banta A. B. Beveridge T. J. Kirshtein J. D. Schouten S. (2006). A ubiquitous thermoacidophilic archaeon from deep-sea hydrothermal vents. Nat. Lett. 442, 444–447 10.1038/nature04921 16871216

[B57] Rogers K. L. Amend J. P. (2005). Archaeal diversity and geochemical energy yields in a geothermal well on Vulcano Island, Italy. Geobiology 3, 319–332 10.1111/j.1472-4669.2006.00064.x

[B58] Schlesner H. Lawson P. A. Collins M. D. Weiss N. Wehmeyer U. Voelker H. (2001). *Filobacillus milensis* gen. nov., sp. nov., a new halophilic spore-forming bacterium with Orn-D-Glu-type peptidoglycan. Int. J. Syst. Evol. Microbiol. 51, 425–431 11324591 10.1099/00207713-51-2-425

[B59] Schloss P. D. Westcott S. L. Ryabin T. Hall J. R. Hartmann M. Hollister E. B. (2009). Introducing mothur: open-source, platform-independent, community-supported software for describing and comparing microbial communities. Appl. Environ. Microbiol. 75, 7537–7541 10.1128/AEM.01541-09 19801464 PMC2786419

[B60] Schrenk M. O. Kelley D. S. Bolton S. A. Baross J. A. (2004). Low archaeal diversity linked to subseafloor geochemical processes at the Lost City hydrothermal field, Mid-Atlantic Ridge. Environ. Microbiol. 6, 1086–1095 10.1111/j.1462-2920.2004.00650.x 15344934

[B61] Seeberg-Elverfeldt J. Schlüter M. Feseker T. Kölling M. (2005). Rhizon sampling of pore waters near the sediment/water interface of aquatic systems. Limnol. Oceanogr. Methods 3, 361–371 10.4319/lom.2005.3.361

[B62] Sievert S. M. Brinkhoff T. Muyzer G. Ziebis W. Kuever J. (1999). Spatial heterogeneity of bacterial population along an environmental gradient at a shallow submarine hydrothermal vent near Milos Island, Greece. Appl. Environ. Microbiol. 65, 3834–3842 10473383 10.1128/aem.65.9.3834-3842.1999 PMC99708

[B63] Sievert S. M. Heidorn T. Kuever J. (2000a). *Halothiobacillus kellyi* sp. nov., a mesophilic obligately chemolithoautotrophic sulfur-oxidizing bacterium isolated from a shallow-water hydrothermal vent in the Aegean Sea and emended description of the genus *Halothiobacillus*. Int. J. Syst. Evol. Microbiol. 50, 1229–1237 10.1099/00207713-50-3-1229 10843067

[B65] Sievert S. M. Kuever J. Muyzer G. (2000b). Identification of 16S ribosomal DNA-defined bacterial populations at a shallow submarine hydrothermal vent near Milos Island (Greece). Appl. Environ. Microbiol. 66, 3102–3109 10.1128/AEM.66.7.3102-3109.2000 10877814 PMC92119

[B64] Sievert S. M. Kuever J. (2000). *Desulfacinum hydrothermale* sp. nov., a thermophilic sulfate-reducing bacterium from geothermally heated sediments near Milos island, Greece. Int. J. Syst. Evol. Microbiol. 50, 1239–1246 10.1099/00207713-50-3-1239 10843068

[B66] Spano A. Gugliandolo C. Lentini V. Maugeri T. L. Anzelmo G. Annarita P. (2013). A novel EPS-producing strain of *Bacillus licheniformis* isolated from a shallow vent off Panarea Island (Italy). Curr. Microbiol. 67, 21–29 10.1007/s00284-013-0327-4 23397221

[B67] Stüben D. Glasby G. P. (1999). Geochemistry of shallow submarine hydrothermal fluids of Paleochori Bay, Milos, Aegean Sea. Explor. Mining Geol. 8, 273–287

[B68] Switzer B. J. Burns B. A. Buzzelli J. Stolz J. F. Oremland R. S. (1998). *Bacillus arsenicoselenatis* sp. nov., and *Bacillus selentireducens* sp. nov.: two haloalkaliphilies from Mono Lake, California that respire oxyanions of selenium and arsenic. Arch. Microbiol. 171, 19–30 10.1007/s002030050673 9871015

[B69] Sylvan J. B. Toner B. M. Edwards K. J. (2012). Life and death of deep-sea vents: bacterial diversity and ecosystem succession on inactive hydrothermal sulfides. mBio 3, 1–10 10.1128/mBio.00279-11 22275502 PMC3262234

[B70] Takai K. Horikoshi K. (1999). Genetic diversity of archaea in deep-sea hydrothermal vent environments. Genetics 152, 1285–1297 10430559 10.1093/genetics/152.4.1285 PMC1460697

[B71] Tarasov V. G. Gebruk A. V. Mironov A. N. Moskalev L. I. (2005). Deep-sea and shallow-water hydrothermal vent communities: two different phenomena? Chem. Geol. 224, 5–39 10.1016/j.chemgeo.2005.07.021

[B72] Tarasov V. G. Propp M. V. Propp L. N. Zhirmunsky A. V. Namsaraev B. B. Gorlenko V. M. (1990). Shallow-water gasohydrothermal vents of Ushishir Volcano and the ecosystem of Kraternaya Bight (The Kurile Islands). Mar. Ecol. 11, 1–23 10.1111/j.1439-0485.1990.tb00225.x

[B73] Valsami-Jones E. Baltatzis E. Bailey E. H. Boyce A. J. Alexander J. L. Magganas A. (2005). The geochemistry of fluids from an active shallow submarine hydrothermal system: Milos island, Hellenic Volcanic Arc. J. Volcanol. Geothermal Res. 148, 130–151 10.1016/j.jvolgeores.2005.03.018

[B74] Varnavas S. P. Cronan D. S. (1988). Arsenic, antimony and bismuth in sediments and waters from the Santorini hydrothermal field, Greece. Chem. Geol. 67, 295–305 10.1016/0009-2541(88)90135-0

[B75] Von Damm K. L. Lilley M. D. Shanks W. C. Brockington M. Bray A. M. O'Grady K. M. (2003). Extraordinary phase separation and segregation in vent fluids from the southern East Pacific Rise. Earth Planet. Sci. Lett. 206, 365–378 10.1016/S0012-821X(02)01081-6

[B76] Wang Q. Garrity G. M. Tiedje J. M. Cole J. R. (2007). Naive Bayesian classifier for rapid assignment of rRNA sequences into the new bacterial taxonomy. Appl. Environ. Microbiol. 73, 5261–5267 10.1128/AEM.00062-07 17586664 PMC1950982

[B77] Wirsen C. (2004). Is life thriving deep beneath the seafloor? Oceanus 42, 1–6 Available online at: http://oceanusmag.whoi.edu/v42n2/wirsen.html

[B78] Zargar K. Conrad A. Bernick D. L. Lowe T. M. Stolc V. Hoeft S. (2012). ArxA, a new clade of arsenite oxidase within the DMSO reductase family of molybdenum oxidoreductases. Environ. Microbiol. 14, 1635–1645 10.1111/j.1462-2920.2012.02722.x 22404962

[B79] Zargar K. Hoeft S. E. Oremland R. S. Saltikov C. (2010). Identification of a novel arsenite oxidase gene, *arxA*, in the haloalkaliphilic, arsenite-oxidizing bacterium *Alkalilimnicola ehrlichii* strain MLHE-1. J. Bacteriol. 192, 3755–3762 10.1128/JB.00244-10 20453090 PMC2897359

